# FRET Monitoring of a Nonribosomal Peptide Synthetase Elongation Module Reveals Carrier Protein Shuttling between Catalytic Domains

**DOI:** 10.1002/anie.202212994

**Published:** 2022-10-26

**Authors:** Jennifer Rüschenbaum, Wieland Steinchen, Florian Mayerthaler, Anna‐Lena Feldberg, Henning D. Mootz

**Affiliations:** ^1^ University of Münster Institute of Biochemistry Corrensstraße 36 48149 Münster Germany; ^2^ Philipps-University Marburg SYNMIKRO Research Center & Faculty of Chemistry Karl-von-Frisch-Straße 14 35043 Marburg Germany

**Keywords:** Conformational Changes, Genetic Code Expansion, HDX-MS, NRPS, Photocaging

## Abstract

Nonribosomal peptide synthetases (NRPSs) employ multiple domains, specifically arranged in modules, for the assembly‐line biosynthesis of a plethora of bioactive peptides. It is poorly understood how catalysis is correlated with the domain interplay and associated conformational changes. We developed FRET sensors of an elongation module to study in solution the intramodular interactions of the peptidyl carrier protein (PCP) with adenylation (A) and condensation (C) domains. Backed by HDX‐MS analysis, we discovered dynamic mixtures of conformations that undergo distinct population changes in favor of the PCP‐A and PCP‐C interactions upon completion of the adenylation and thiolation reactions, respectively. To probe this model we blocked PCP binding to the C domain by photocaging and triggered peptide bond formation with light. Changing intramodular domain affinities of the PCP appear to result in conformational shifts according to the logic of the templated assembly process.

## Graphical abstract

Translocation of the peptidyl carrier protein (PCP) between two catalytic domains in nonribosomal peptide synthetases (NRPSs) has been studied for the first time. Monitoring PCP interaction with A and C domains during catalysis using two FRET sensors of an elongation module revealed conformational population shifts that correlate with the directionality of synthesis. The first light‐controlled NRPS was created to further probe and support this model.

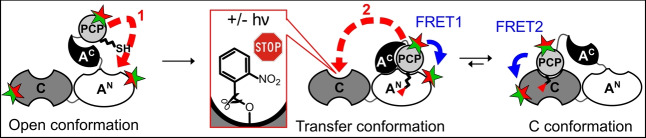

## Introduction

**Figure 1:**
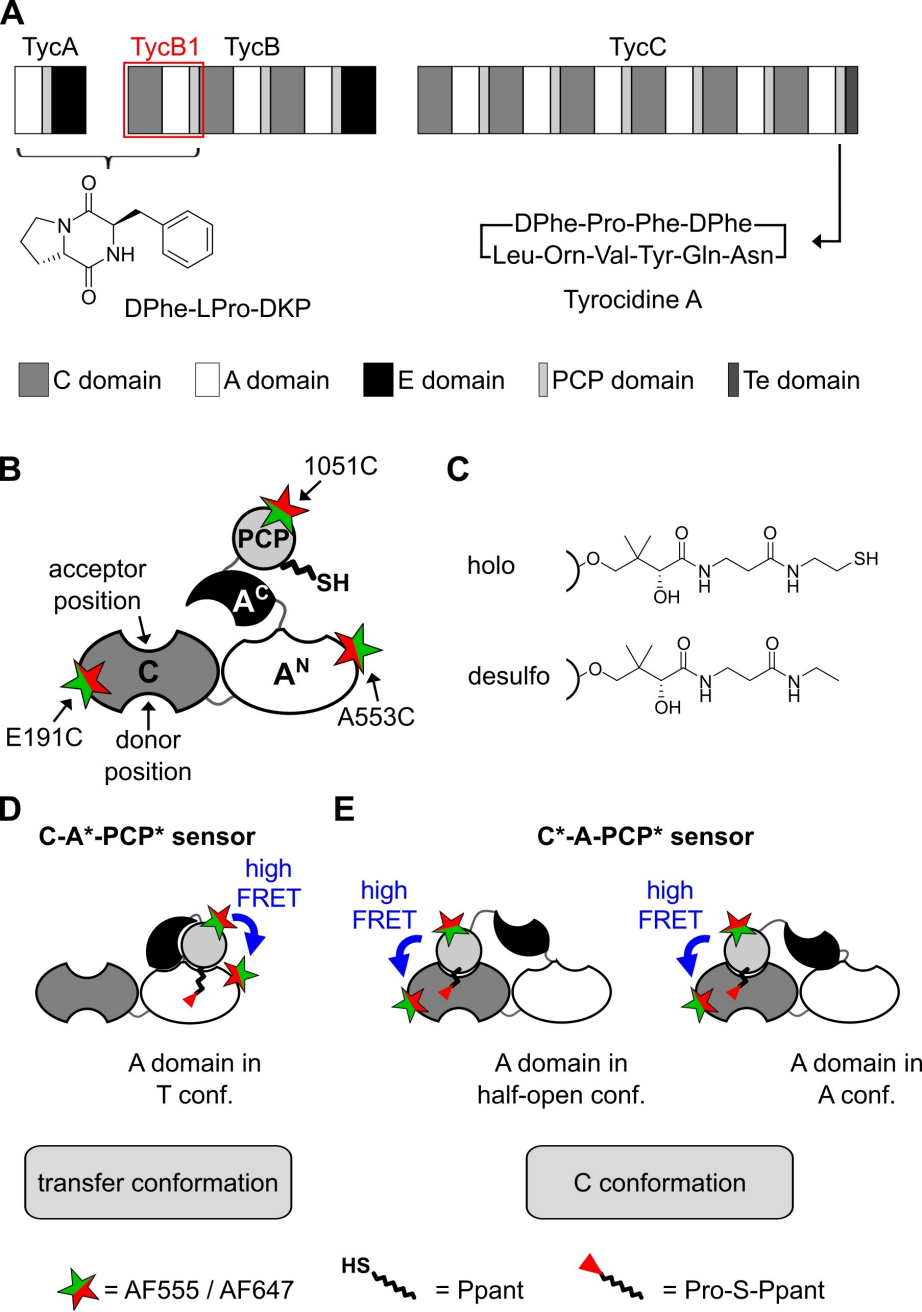
The tyrocidine NRPS and the derived TycB1 FRET sensors. A) The three‐enzyme, ten‐module tyrocidine NRPS. The TycB1 module is boxed in red. TycA and TycB1 can form the D‐Phe‐L‐Pro‐diketopiperazine (D‐Phe‐L‐Pro‐DKP) shunt product. B) Domain composition of the L‐Pro‐specific TycB1 module with fluorophore attachment positions used in this study. C) Chemical structures of holo‐ and desulfo‐Ppant prosthetic groups. D), E) Design of C‐A*‐PCP* and C*‐A‐PCP* sensors intended to report on either the transfer conformation or the C conformation, respectively. Relative to C and A^N^ domains, the A^C^ and PCP domains are highly mobile. Note that in the C conformations shown in (E), specified by the PCP‐C interaction, the A domain is known to be capable of adopting either a half‐open (left panel)^[3a]^ or the A conformation (right panel).^[3b]^

Nonribosomal peptide synthetases (NRPSs) are involved in the biosynthesis of many pharmaceutically important bioactive peptides. The engineering of their modular structure holds great potential for the generation of new products.^[1]^ NRPSs are composed of modules that are responsible for the incorporation of a dedicated amino acid into the growing peptide chain. Typically, the arrangement of modules is co‐linear with the synthesized peptide,^[2]^ reminiscent of an assembly line, in which activated amino acyl building blocks and peptidyl intermediates are covalently bound and shuttled from module to module. A minimal elongation module is composed of an adenylation (A) domain for ATP‐dependent substrate activation, a peptidyl carrier protein (PCP) or thiolation (T) domain for acyl group binding as a 4′‐phosphopantetheine (Ppant) thioester, and a condensation (C) domain for peptide bond formation. Further domains are utilized for optional tailoring and release of the product from the NRPS template (Figure 1A).

One of the intriguing questions about the protein‐templated NRP biosynthesis is how the catalytic and conformational interplay of NRPS domains and modules is facilitated in space and time to result in the directional synthesis of a specific product. NRPSs domains and modules are known to undergo some significant conformational changes. Static snapshots in crystal structures have shown that the PCP has to translocate over long distances to reach the different catalytic domains.^[3]^ Furthermore, the A domain undergoes a domain alternation mechanism by closing and reconfiguring its large A^N^ and small A^C^ subdomains relative to one another along the adenylation and thiolation reactions of the aminoacylation pathway, thereby adopting at least an open (O), an adenylation (A), and a thiolation (T) conformation.^[4]^ However, in‐solution techniques are required to observe all relevant conformations and to understand how catalysis is linked to conformational dynamics.

We have previously explored intramolecular FRET spectroscopy and HDX‐MS analysis to study conformational changes in NRPSs.^[5]^ A FRET sensor was designed based on the excised A‐PCP didomain unit from the GrsA initiation module (with A‐PCP‐E domain composition). It helped reveal that the interacting A and PCP domains adopt a mixture of conformations in a conformational equilibrium. Shifts in the population of different conformation(s) were observed when triggering different states of the enzyme, i.e., in presence of different ligands or substrates.^[5]^ In particular, addition of substrates ATP and amino acid triggered a conformational shift in favor of the transfer conformation,^[5]^ in which the PCP productively binds to the A domain. Furthermore, we proposed the Ppant‐threading conformation as intermediary conformation in the A domain alternation and detected a rate‐determining conformational change.^[5b]^ The preferential binding of the aminoacylated PCP to the A domain in the A‐PCP FRET sensor was surprising.^[5a]^ While this observation makes biochemical sense as a mechanism to shield the reactive thioester from the solvent and other undesired nucleophiles, favoring this product‐inhibited complex is counterintuitive with regard to the directionality of the NRPS assembly line as it does not explain how the aminoacylated PCP is further processed. Since the A‐PCP FRET sensor lacked other domains for the downstream synthesis logic, their influence on the subsequent shuttling of the aminoacylated PCP has remained unknown so far. A more holistic description using a multidomain NRPS is required to understand how directional peptide synthesis is achieved.

The C domain is part of an elongation module (domain composition: C‐A‐PCP). It binds its aminoacyl‐S‐PCP_(*n*)_ substrate as extender unit at its acceptor site and the incoming upstream aminoacyl‐ or peptidyl‐S‐PCP_(*n*−1)_ from the preceding module at its donor site.^[6]^ According to an earlier hypothesis, binding of aminoacyl‐PCP at the acceptor position serves as a capturing event to prevent premature translocation to the downstream module and thereby potential mis‐initiation of peptide synthesis at internal modules.[^6^, ^7^] The conversion of elongation modules into initiation modules by deleting their C domain provided indirect support for this idea.^[6b]^ However, such truncation experiments are complicated by the potentially drastic and unpredictable impact on the activity of the remaining domains.^[8]^ It remains unknown whether aminoacyl thioester formation indeed triggers a conformational shift to a preferred PCP binding at the acceptor position of the C domain. Thus, studying the influence of a C domain on the conformational dynamics is key to understand the domain interplay and how aminoacylation primes an elongation module for the next catalytic step.

In this study, we addressed the question how a PCP shuttles between the A and C domains as competing binding partners. We designed a set of novel FRET sensors of an elongation module to report on the PCP interactions with both the A and the C domain. Corroborated by an HDX‐MS analysis and a novel approach to control the PCP‐C interaction by light, we identified a conformational shift of the aminoacylated module towards the C conformation. This study expands our understanding of the dynamics of conformational changes in NRPS during catalysis.

## Results and Discussion

### Design of FRET Sensors of an NRPS Elongation Module

We aimed to study conformational changes within an elongation module (C‐A‐PCP domains) that are associated with the aminoacylation reaction. We envisaged FRET spectroscopy as an in‐solution technique to unravel domain interactions and selected the second module of the tyrocidine NRPS, TycB1, for our studies (Figure 1A).^[9]^ Crystal structures of homologous NRPSs suggested that a platform of the C domain and the A^N^ subdomain organize two well‐separated binding sites for the PCP domain at the A and C domains (Figure 1B).[^3^, ^10^] The mobility of the A^C^ subdomain relative to the A^N^ subdomain and the flexible A^C^‐PCP linker facilitate PCP translocation between the two active sites (Figure 1D–E).

To monitor the interaction of the PCP domain with either of the two catalytic domains, we created two different types of novel FRET sensors (Tab. S1–3, Figure S1). We designed a C‐A*‐PCP* sensor to report on the transfer conformation, representative for the PCP‐A interaction (Figure 1D), and a C*‐A‐PCP* sensor to be specific for the C conformation, representative for the PCP‐C interaction (Figure 1E). A construct lacking the C domain served as control for the influence of the C domain (A*‐PCP* sensor). To this end, we removed eight out of the 10 native cysteines in the 1045 aa TycB1 construct by replacement with serine or alanine, resulting in no detectable loss of activity in the aminoacylation reaction (Figure S2). We showed that the remaining C657 and C662 were unreactive in the following bioconjugation reactions (Figure S3). Two appropriately positioned cysteines were then introduced for stochastic attachment of synthetic donor and acceptor fluorophores. A short sequence with 1051C was appended at the C terminus of the PCP in both sensor constructs. As the second position we chose A553C in the A^N^ subdomain for the C‐A*‐PCP* sensor and E191C in the C domain for the C*‐A‐PCP* sensor (Figure 1 and Table S2). These positions were selected based on their proximity in the range of the Förster radius in structural models of the transfer[^3b^, ^4a^, ^5a^, ^11^] and C conformations, respectively.^[3]^

**Figure 2:**
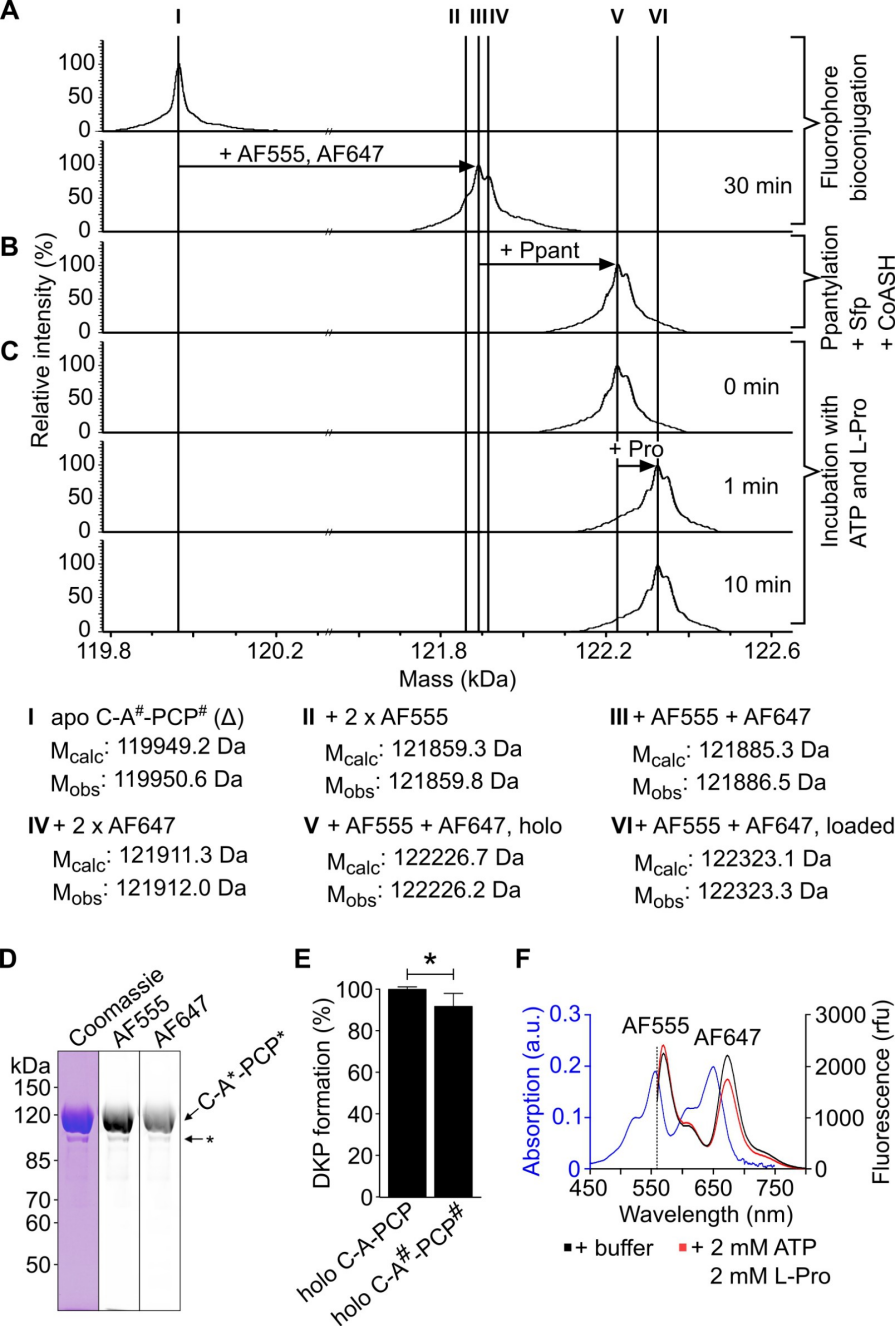
Characterization of the TycB1 C‐A*‐PCP* FRET sensor. A), B), C) ESI‐MS analysis of fluorophore bioconjugation, Ppantylation and aminoacylation reaction. D) SDS‐PAGE analysis of AF555 and AF647‐conjugated C‐A*‐PCP* by Coomassie‐staining and in the respective fluorescent channels. The asterisk denotes minor protein contamination. E) D‐Phe‐L‐Pro‐DKP formation (Figure S5) with holo‐GrsA (after 30 min). Shown are the mean ±SD of three independent replications. * indicates *p*≤0.05 (Student's t‐test). F) Absorption and fluorescence spectra, the latter with and without substrates, of holo‐C‐A*‐PCP* sensor shows substrate‐dependent FRET response.

The protein mutants were expressed in *E. coli* cells as apo proteins and affinity‐purified (termed C‐A^#^‐PCP^#^ and C^#^‐A‐PCP^#^ in unconjugated form). Thiol‐bioconjugation with maleimide dyes Alexa Fluor®555 (AF555) and Alexa Fluor®647 (AF647) as FRET donor and acceptor dyes,^[12]^ respectively, resulted in the doubly modified conjugates with a high yield of the desired species containing both types of dye (species III, V and VI in Figure 2A–C and S4A–C). Finally, the proteins were converted into the catalytically active holo‐proteins (holo‐sensors) by using the 4′‐Ppant‐transferase Sfp and coenzyme A (CoA) (Figure 2B and S4B).^[5]^ Additionally, we prepared for each sensor type a desulfo‐variant posttranslationally modified with desulfo‐CoA (Figure 1C), thus handicapped in forming the aminoacyl thioesters.

The TycB1 sensors were biochemically characterized and shown to exhibit enzymatic activities of virtual wild‐type levels. Both the holo‐C‐A*‐PCP* and holo‐C*‐A‐PCP* sensors were indistinguishable from the unmutated holo‐TycB1 module in the virtually quantitative ATP‐dependent aminoacylation reaction with the substrate L‐Pro, which rapidly occurred within ≤1 min (Figure 2C and S4C).^[9b]^ Furthermore, the mutated constructs showed only a minor reduction in the D‐Phe‐L‐Pro forming elongation reaction with the compatible initiation module GrsA (Figure 2E and S4E). In this assay, the dipeptide autocatalytically cleaves itself as cyclic diketopiperazine (DKP) to allow for enzymatic turnover.[^9b^, ^13^] Thus, the novel FRET sensors set the stage to investigate the three‐domain interplay in an elongation module.

### A Transient Conformational Shift towards the Transfer Conformation

**Figure 3:**
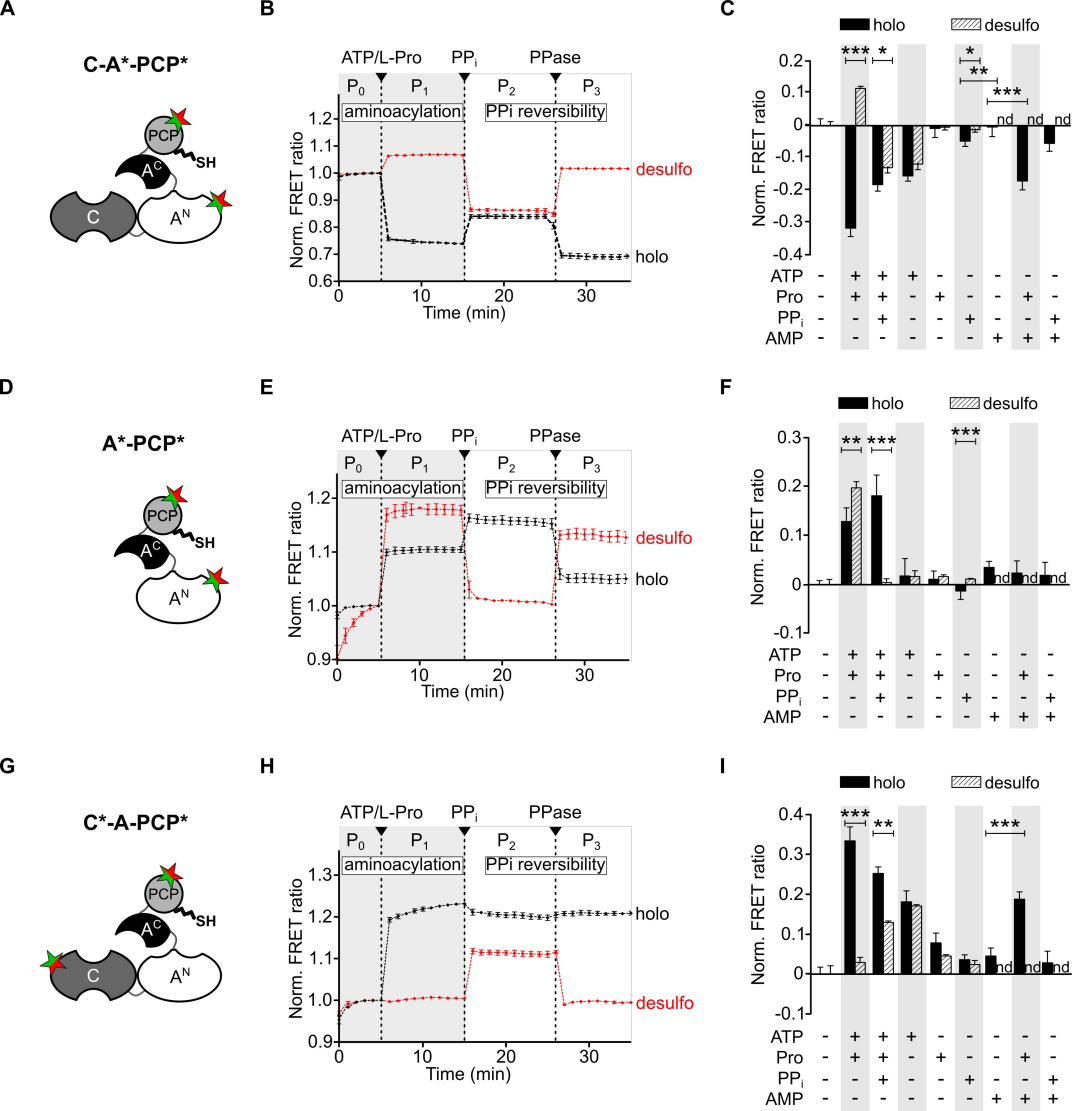
Conformational changes of TycB1 FRET sensors in correlation to ligand binding and catalysis. A), D), G) Respective sensor constructs. Each sensor was investigated in holo‐ and desulfo‐forms. B), E), H) Time‐dependent FRET measurements. ATP, L‐Pro (each 2 mM), PP_I_ (10 mM) and PPase (2 U) were added at the indicated time points. C), F), I) FRET ratios after incubation for 30 min with indicated ligands. FRET ratios (AF647/AF555) are normalized to the buffer control before addition of ligands. In the right panel the buffer control was set to zero for easier comparison. nd=not determined. All FRET data shown are from at least three biological repeats with three independent measurements. Note that time‐resolved (in B, E, H) and endpoint FRET measurements (in C, F, I) were measured with different instruments, resulting in slightly different absolute FRET values but showing similar qualitative signals. *p≤0.05,**p≤0.01,***p≤0.001 (Student's *t*‐test).

To investigate the interaction between PCP and A domains in the TycB1 elongation module, we performed time‐dependent bulk FRET experiments with the holo‐C‐A*‐PCP* sensor. The formation of the prolyl‐TycB1 thioester triggered by addition of the substrates ATP and L‐Pro (see above; Figure 2C) correlated with the rapid jump of the FRET ratio to the plateau P1 (Figure 3B, black line). We calculated the FRET ratio as the quotient of the bulk acceptor and donor intensities (*I*_A_/*I*_D_) and normalized it to the respective ratio of the buffer‐only conditions in the absence of substrates (plateau P0; compare maxima of red and black curves in Figure 2F). Figure 3C shows that the maximal FRET response was obtained for ATP+L‐Pro, suggesting maximal conformational changes caused by the aminoacylation reaction. Not surprisingly, some other conditions with single substrates or ligands, or combinations thereof, also resulted in FRET ratio changes as well, although less pronounced, and thus indicated conformational changes. These cases pertained to ATP, L‐Pro+AMP, and the combination of ATP+L‐Pro+PP_i_, and will be discussed in the “fingerprint” section (see below).

According to the sensor design, the observed FRET ratio decrease to plateau P1 in presence of ATP and L‐Pro (P1<P0; Figure 3B, black line) suggested a conformational shift in the averaged protein population that resulted in a larger distance between the labeled PCP and A domains. Thus, the transfer conformation would be depopulated to a certain degree. Interestingly, this FRET response is qualitatively opposite to the FRET ratio *increase* we had previously observed for the A‐PCP didomain FRET sensor of the GrsA initiation module in response to ATP and amino acid substrate addition, as mentioned in the introduction.^[5]^ Control experiments with a newly designed GrsA didomain sensor ruled out that the nature and positions of the fluorophores accounted for these different FRET responses (Figure S6). These findings thus suggested an influence of the C domain.

To further corroborate the TycB1 holo‐C‐A*‐PCP* sensor as the intended reporter of the transfer conformation we carried out several controls with partially impaired variants. The desulfo‐C‐A*‐PCP* sensor with the lacking thiol moiety in the Ppant group cannot form the prolyl‐thioester. Therefore, added substrates would only lead to formation of the Pro‐AMP intermediate and be expected to lock the protein in the transfer conformation prior to the thiolation reaction. Consistent with this notion, addition of ATP and L‐Pro led to a FRET ratio *increase* to plateau P1, albeit with relatively smaller amplitude (Figure 3B, red line). Using the TycB1 control constructs lacking the C domain, we also observed the expected FRET ratio *increases* for holo‐A*‐PCP* and desulfo‐A*‐PCP* sensors (Figure 3D–F and Figure S7), qualitatively comparable to the previously reported GrsA A‐PCP sensors (Figure S6).^[5a]^ These FRET responses are consistent with conformational shifts towards the post‐transfer conformation caused by aminoacyl‐S‐PCP binding to the A domain in case of the holo‐sensor, and towards the pre‐transfer conformation in the context of the desulfo‐sensor, respectively. Together, the controls validated the TycB1 C‐A*‐PCP* sensors as being reporters for the transfer conformation of the PCP‐A interaction.

Importantly, in the context of the C‐A‐PCP elongation module, the distinct FRET responses of the different sensors can be put in a sequence that indicated a transient conformational shift associated with the aminoacylation reaction. In agreement with the NRPS synthesis logic, first the added ATP and L‐Pro induce PCP binding to the A domain in a transfer conformation for the thiolation reaction (observed with the desulfo‐C‐A*‐PCP* sensor; P1>P0). Second, upon completed aminoacyl thioester formation, the transfer conformation is depopulated, i.e. PCP detaches from the A domain (because P1<P0 for holo‐C‐A*‐PCP*; further indicating that the transfer conformation becomes even less populated than in the absence of substrates, i.e., the transfer conformation must show a significant population in this basal state).

### A Conformational Shift in Favor of the C Conformation Occurs after PCP Aminoacylation

We next asked whether the observed depopulation of the transfer conformation after completion of the aminoacylation reaction correlated with a higher population of the C conformation. To address this question, we turned to the holo‐C*‐A‐PCP* sensor, designed to be specific for the PCP‐C interaction (Figure 3G–I). If the PCP is shuttled between the A and C domains, this sensor should give a FRET output reciprocal to that of the holo‐C‐A*‐PCP* sensor. Indeed, such a response was observed when triggering the aminoacylation reaction of the holo‐C*‐A‐PCP* sensor. We monitored the FRET ratio to *increase* to plateau P1, indicative of increased proximity between the Pro‐S‐Ppant‐PCP and the C domain (Figure 3H, black line). This finding suggested an increased population of the C conformation compared to the uncharged protein in the absence of substrates (plateau P0). Again, controls using single substrates and other combinations suggested less pronounced conformational changes also for certain other conditions (Figure 3I), that are discussed in the next section. Of note, shuttling of the aminoacylated PCP to the C domain makes biochemical sense as a conformational priming step for the condensation reaction, the next step in the logic of the assembly line mechanism.

Another insight could be gained from an investigation of the desulfo‐C*‐A‐PCP* sensor. This protein is incapable of the thiolation reaction. Following formation of Pro‐AMP it therefore traps the PCP at the A domain in transfer conformation, just like the biochemically identical desulfo‐C‐A*‐PCP* sensor, which showed the conformational shift in favor of the transfer conformation (P1>P0; Figure 3B, red line). Interestingly, desulfo‐C*‐A‐PCP* did not show a reciprocal response with P1<P0 under these conditions. Instead, we observed virtually P1=P0 for this sensor (Figure 3 H, red line). These findings indicate that the initial, substrate‐induced translocation of the desulfo‐PCP to the A domain does not occur at the expense of the C conformation, or at best marginally. In other words, and if also true for the chemically slightly different holo‐PCP proteins, the transient increase of the transfer conformation to catalyze the aminoacylation reaction would be fueled from other conformations.

### FRET Sensors of Different Modules Show “Fingerprint” Responses to Some Ligands

As mentioned above, three other ligand additions also resulted in pronounced FRET ratio changes for some of the sensor proteins, specifically 1) ATP as a single substrate, 2) L‐Pro+AMP, and 3) ATP+L‐Pro+PP_i_ (Figure 3). These findings suggested that binding of these ligands triggered conformational changes, as interpreted in the following.

The first two conditions represent ligand combinations expected to mostly favor the closed A conformation of the A domain. Therefore, they should concomitantly disfavor the transfer conformation, which requires the A domain in T conformation, and which shows a basal population in the absence of ligands (see above). As a consequence, PCP is released from the A domain and therefore more likely to bind to the C domain, thus favoring the C conformation. These considerations explain the decreased and increased FRET ratio responses of the C‐A*‐PCP* and C*‐A‐PCP* sensors, respectively. In contrast, the A*‐PCP* sensors showed no significant responses to the first two conditions. This latter finding may be explained by the missing C domain, because without the C domain also the C conformation is removed from the conformational space of these sensors. The remaining conformations like open, A and transfer conformations are thus likely to adopt altered populations compared to the C‐A‐PCP proteins, both in the absence and presence of ligands, resulting in different FRET sensor readouts. In support of these considerations, an altered conformational equilibrium in the absence of the C domain is obvious from the experimental data for the desulfo‐A*‐PCP* sensor. Here, P2 is about the same as P0, whereas P2<P0 for desulfo‐C‐A*‐PCP*. Considering that excess PP_i_ disrupts the transfer conformation, this finding suggests that desulfo‐A*‐PCP* does not populate the transfer conformation in the absence of substrates, in contrast to desulfo‐C‐A*‐PCP*.

In the third condition, PP_i_ is present at excess concentrations when the enzyme has the L‐Pro‐S‐Ppant thioester. As reported previously, PP_i_ binding reverses the domain alternation mechanism and disfavors the transfer conformation by competing with a conserved Glu‐Arg salt bridge between the A^C^ and A^N^ subdomains (E761 and R873 in TycB1).[^4^, ^5^] Removal of the excess PP_i_ by adding pyrophosphatase (PPase) showed the reversibility of this effect (see plateau changes from P2 to P3 in time‐courses of Figure 3B, E, H and Figure S6B & S8). In case of the desulfo‐sensors, PP_i_ binding results in a detachment of the PCP from the A domain, which in turn can increase PCP binding to the C domain (explaining why P2< P1 for desulfo‐C‐A*‐PCP* and desulfo‐A*‐PCP*, and P2> P1 for desulfo‐C*‐A‐PCP*). In cases of the holo‐sensors, the PCP may still bind to the A domain owing to the affinity of the aminoacyl thioester to the active site. However, it can do so only in the postulated, half‐open Ppant‐threading conformation[^5b^, ^14^] that can concomitantly accommodate the bound PP_i_ (Figure S9). The observation of a FRET ratio increase (P2> P1) in these cases for the transfer conformation sensors (holo‐C‐A*‐PCP* and holo‐A*‐PCP*) and of a decrease for the C conformation sensor (P2< P1 for holo‐C*‐A‐PCP*) suggests a higher stability or higher FRET signaling capability of the Ppant‐threading conformation compared to the transfer conformation. The TycB1 sensors labeled at PCP and A domains thus report on both the transfer and the Ppant threading conformations, which is consistent with the overall quite similar positioning of the two domains in these conformations.[^5b^, ^14^]

In summary, all FRET ratio changes of the TycB1 sensors can be accounted for with consistent models of their conformational changes. However, apart from the focus on the PCP shuttling logic, this work also revealed some clearly discernable FRET responses of the TycB1 sensors when compared to the previously investigated A‐PCP unit of the GrsA initiation module.^[5]^ Examples are the responses to some ligands like AMP and the above‐discussed Ppant‐threading conformation. We performed control experiments shown in Figures S6 and S7 to rule out slight differences in the sensor design as alternative underlying cause. We assume that next to differences in relative stabilities of conformations of an A‐PCP didomain unit also the slight variability *within* these conformations between different modules can influence the FRET read‐out (see ref. [15] and references therein to showcase the variability of the transfer conformation). We refer to these differences as the “fingerprint” of NRPS domain interactions. Our work suggests the power of FRET as in‐solution technique to reveal such differences in the conformational fine‐tuning.

### HDX‐MS Analysis Confirms Conformational Shift

We performed HDX‐MS experiments to independently investigate the proposed conformational shift towards the C conformation upon aminoacylation of TycB1. The HDX rate depends on the accessibility of the amide protons within the peptide chain. Differences in HDX between two experimental settings may reflect perturbations in conformation. Briefly, we incubated wildtype holo‐TycB1, pre‐incubated for 20 min with or without ATP/L‐Pro, in D_2_O‐containing buffer and stopped the HDX reaction after various time periods by acidification. Following pepsin digestion, a total of 323 peptides was identified by MS, covering 93.8 % of the TycB1 sequence with 3.85‐fold redundancy per amino acid (Supporting Information Dataset).

**Figure 4:**
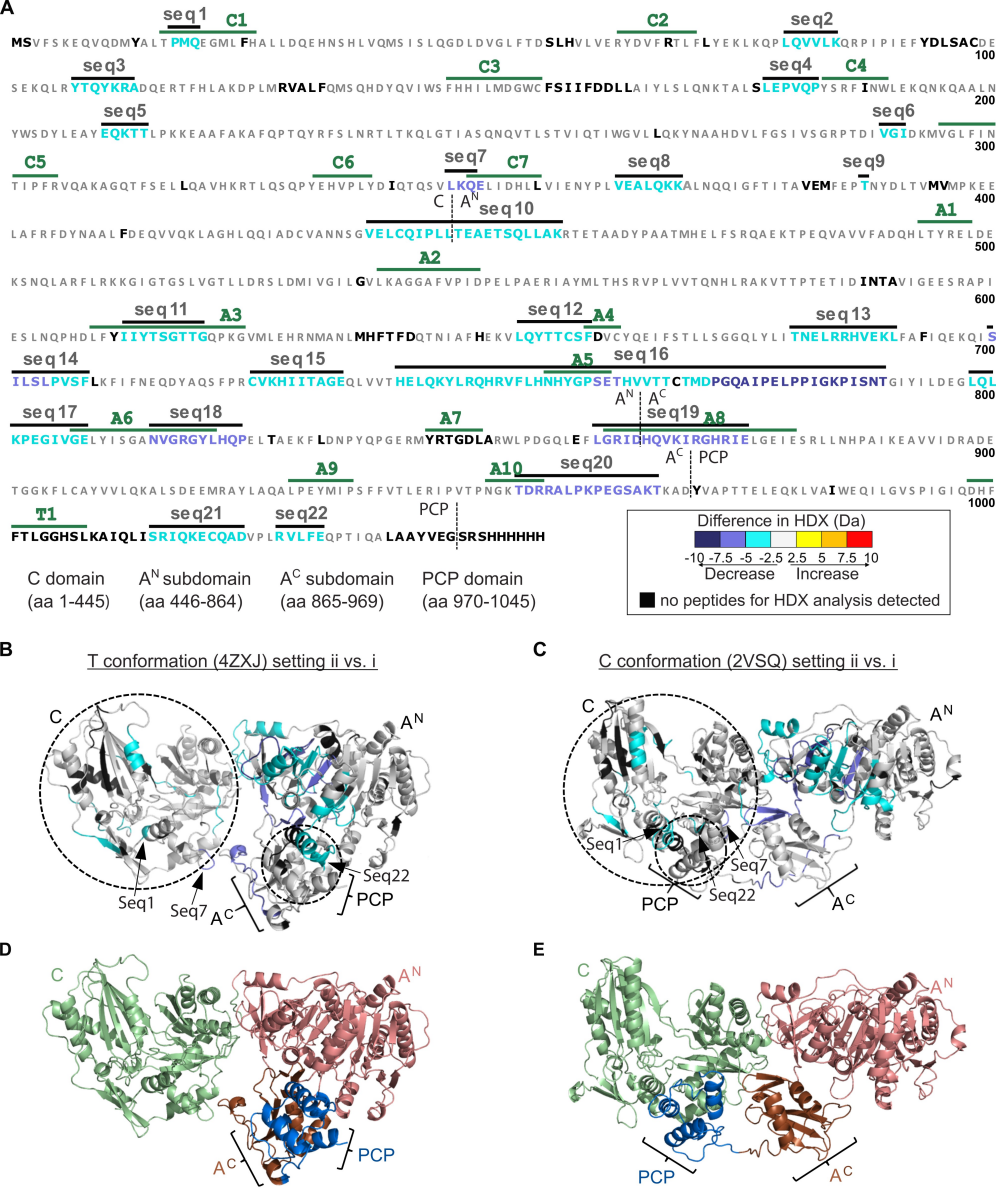
HDX‐MS analysis of wildtype TycB1 (holo‐C‐A‐PCP). HDX was compared between two experimental settings: i) buffer control, and ii) 30 min after addition of ATP/L‐Pro (each 1 mM). A decrease of HDX observed for (ii) compared to (i) represents a shielding resulting from the substrates and the aminoacylated PCP. A) Amino acid sequence of the protein with indicated conserved motifs (marked in green). Seq1‐seq22 denote sequence stretches with altered HDX rates. B), C) Seq1‐seq22 and residues lacking in HDX analysis (black) mapped onto Phyre2‐modeled structures of TycB1in the transfer and C conformations using the pdb entries 4ZXJ and 2VSQ, respectively.^[16]^ Seq1, 7 and 22 assumed to be involved in the PCP‐C contact are highlighted. D), E) Visualization of domain boundaries in the same structural models using a separate color code. Structural images were created with PyMol. The HDX color legend applies to panels (A)–(C).

We observed differences in HDX between the two settings for 22 sequence stretches, all of which became less accessible for HDX in the presence of ATP/L‐Pro compared to the buffer control without substrates (Figure 4A and Figure S10–S12). Figure 4B shows these HDX changes mapped onto structural models of the C‐A‐PCP domains of TycB1 in C and in transfer conformations (modeled using Phyre2 into the pdb entries 2VSQ and 4ZXJ, respectively). Sequence stretches seq10 to seq20 are located in the A domain, of which seq10 and seq17 are in the linker and interface regions between C and A domains, suggesting an altered interdomain contact. The remaining sequence stretches in the A domain are mostly centered around the active site, consistent with a substrate‐induced closure of the A^N^ and A^C^ subdomains. Notably, both the A8 and A10 motifs^[1a]^ were among the protected sequences. These motifs are located on opposite sides of the mobile A^C^ subdomain and represent the key catalytic motifs for the A domain's T and A conformations, suggesting that both conformations are simultaneously favored compared to the buffer condition. This finding further supports the idea^[5b]^ of dynamic conformational mixtures as a feature of NRPS A‐domains.

Sequence stretches seq1 to seq9 are located in the C domain and are mostly centered around the active site motif HHxxxDG (motif C3),^[1a]^ which is located at the bottom of the cleft between two structurally symmetrical N‐ and C‐lobes, or are found in latch and floor loops that connect the two lobes.^[17]^ It has previously been speculated that a closing motion of the lobes is involved in catalysis of the C domain,^[18]^ a hypothesis that would thus be supported by our findings. No altered HDX was apparent for the C3 motif in the N‐lobe, presumably because this area intrinsically exhibited very low HDX (less than 10 % under our conditions), in contrast to the β‐sheets located in the C‐lobe opposite to C3 (seq7 to seq9). This latter region has been implicated in forming the binding pocket for the side chain of the Ppant‐bound aminoacyl moiety, thus the proposed adoption of the C conformation would be a plausible explanation for the observed changes in the HDX‐MS analysis.^[17b]^ Furthermore, seq1 and seq7 of the C domain as well as seq22 of the PCP domain appear to encompass the contact interface between these two domains and the Ppant prosthetic group (Figure 4). Their shielding in HDX thus provides independent support for our FRET‐derived model of a conformational shift towards the C conformation upon aminoacylation.

### A Light‐Activatable Elongation Module to Probe the Conformational Shift Model

**Figure 5:**
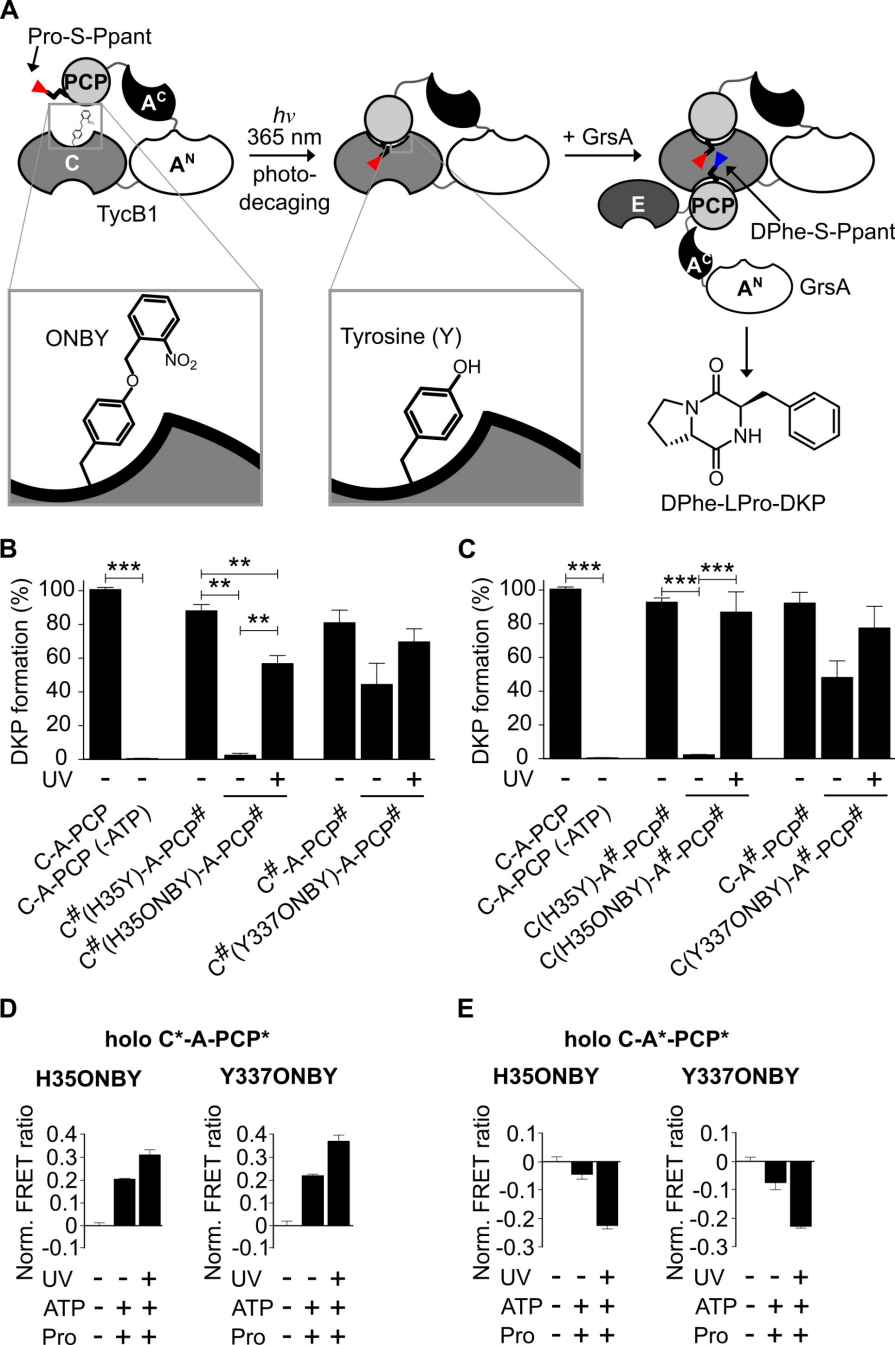
Light‐control of the PCP‐C interaction and peptide formation. A) ONBY‐mediated photocaging of the C domain acceptor site to block PCP binding until decaging with UV light of 365 nm. B), C) D‐Phe‐L‐Pro‐DKP formation (within 30 min) of indicated holo‐TycB1 variants with wildtype GrsA. Shown are the mean ± SD of three independent replications. ** indicates *p*≤0.01, *** indicates *p*≤0.001 (Student's *t*‐test). D), E) FRET ratio analysis of the indicated ONBY‐caged sensors, derived from C*‐A‐PCP* and C‐A*‐PCP*, respectively. FRET ratios (AF647/AF555) were normalized to the buffer control before UV irradiation and addition of ATP and L‐Pro (2 mM each). UV light was applied for 10 s. Data shown are from three biological replicates, each with three independent measurements.

We next sought to probe our model by specifically and reversibly perturbing the system on the conformational level and by monitoring this artificial control by the FRET readout. We envisioned to sterically block the PCP entry to the C domain's active site with a photolabile caging group, thereby preventing or reducing the ability of the elongation module to adopt the C conformation required for dipeptide formation with an incoming initiation module (Figure 5A). Removal of the caging group by light should reconstitute the conformational equilibrium and allow peptide bond formation to occur in presence of the partner module.

To identify suitable positions for the photocage group, we modeled TycB1 onto known crystal structures that show the PCP bound to the acceptor position of the C domain (Figure S13A & B).[^3b^, ^19^] We chose H35 and Y337 to incorporate ortho‐nitrobenzyl‐tyrosine (ONBY) by the genetic code expansion technology.^[20]^ We found both these ONBY‐mutations to clearly impair the D‐Phe‐L‐Pro‐DKP dipeptide formation with the GrsA initiation module. The unlabeled holo‐C‐A^#^‐PCP^#^(ONBY) mutants showed a relative reduction in activity of about 98± 0.2 % and 52± 10 %, respectively, and similar values were obtained for the mutants of holo‐C^#^‐A‐PCP^#^ (Figure 5B & C, Figure S14; the H35Y mutation had only a minor impact). Irradiation of the proteins with UV light (365 nm, 8 W, 10 s) to remove the photocage group (Figure S15) efficiently restored the ability to catalyze dipeptide formation on average to around 80 % (Figure 5B & C, Figure S14). These results represent the first example of a light‐activatable NRPS.

We then investigated whether this activity control occurred by the intended conformational pertubations. We observed for both holo‐C*‐A‐PCP*(ONBY) sensors under aminoacylation conditions with ATP and L‐Pro a FRET ratio increase upon UV irradiation (Figure 5D), consistent with our design idea to disfavor the C conformation by the ONB group. We further hypothesized that the decreased population of the C conformation in the presence of the ONB should result in increased population of one or more of the other conformations within the conformational equilibrium. Indeed, using the respective two holo‐C‐A*‐PCP*(ONBY) sensors reporting on the transfer conformation, we observed in the presence of ATP and L‐Pro a FRET ratio *decrease* upon treatment with 365 nm. This finding was consistent with an ONB‐caused increased population of the transfer conformation before the light‐induced restoration of the native conformational equilibrium under these conditions (Figure 5E).

Finally, these findings provided even further corroboration of our FRET sensor design and associated conformation assignments.

## Conclusion

We report the first in‐solution conformational analysis of a full‐length NRPS module under catalytic conditions to reveal the multidomain interplay. The investigated TycB1 construct is a canonical elongation module composed of C‐A‐PCP domains. To investigate the interactions of the PCP domain with the A and C domains, we designed two sets of FRET sensors to reciprocally report on the proximity of the respective protein pairs in bulk FRET experiments. With further corroboration from HDX‐MS measurements and defined pertubations using steric blocking with photolabile caging groups, we could correlate the FRET sensor read‐outs with conformational changes and interpret the data. Previous findings from a simpler didomain A‐PCP sensor showed that several, if not all, possible conformations can exist in a dynamic mixture^[5]^ and the present work further supports this notion for the C‐A‐PCP module.^[5]^

In general, one would assume that substrate binding or a catalytic step would trigger a conformational shift towards the conformation for the next reaction to be catalyzed. However, in principle it would also be conceivable that the required conformation was dynamically populated already beforehand at sufficient or even dominating levels, in which case only a marginal or no overall such conformational shift would be observable in bulk experiments. In the present work on the C‐A‐PCP elongation module we unraveled two conformational shifts associated with the two‐step aminoacylation reaction that indeed were consistent with the expected NRPS logic. The transfer conformation of the PCP‐A interaction was favored upon formation of the aminoacyl adenylate and subsequently the C conformation of the PCP‐C interaction was favored upon formation of the aminoacyl‐S‐Ppant‐PCP thioester. Aminoacylation of the PCP appears to increase its affinity for the C domain's acceptor position.

Our findings of the conformational shift towards the C conformation also provide direct experimental support for the hypothesis that the interaction between the C domain and aminoacylated PCP helps to prevent mis‐initiation at internal modules, as this binding event would disfavor the premature translocation of the PCP to the donor site of the downstream C domain. However, to fully understand this control of directionality in an elongation module, the downstream C domain of the following module as the remaining interaction partner of the PCP must be included in future experimental designs.

We could further refine our proposal of fine‐tuned affinities of the aminoacylated PCP by obstructing the entry site at the C domain with a sterically demanding photolabile caging group and thereby modulating the affinity of the PCP‐C interaction. Using FRET measurements, we confirmed the decreased binding of the aminoacylated PCP at the blocked C domain. Concomitantly, we could monitor an increased population of the transfer conformation, in which the aminoacylated PCP binds to the A domain. This finding demonstrates that the A domain retained the affinity for the aminoacyl‐PCP. The emerging model of shuttling the aminoacylated PCP between two competing catalytic domains based on affinities thus appears to be self‐sufficient in the sense that it does not require additional allosteric communications between the domains, although we cannot rule these out at present as an additional layer of regulation.

More generally, our insights highlight the importance of the domain‐domain interactions when aiming to construct hybrid NRPS by domain or module swapping experiments. They may help to explain why such experiments can fail and how they could be rescued by optimizing domain contacts.^[21]^ A detailed picture is now emerging of the conformational changes associated with catalysis in selected NRPS constructs of increasing complexity.

Finally, our novel design of a photoactivatable NRPS assembly line may spur novel applications in synthetic biology approaches.

## Conflict of interest

The authors declare no conflict of interest.

1

## Supporting information

As a service to our authors and readers, this journal provides supporting information supplied by the authors. Such materials are peer reviewed and may be re‐organized for online delivery, but are not copy‐edited or typeset. Technical support issues arising from supporting information (other than missing files) should be addressed to the authors.

Supporting Information

## Data Availability

The data that support the findings of this study are available from the corresponding author upon reasonable request.
