# Supplementary material for: FRET Monitoring of a Nonribosomal Peptide Synthetase Elongation Module Reveals Carrier Protein Shuttling between Catalytic Domains
Source: Angew Chem Int Ed Engl. 2022 Oct 26;61(48):e202212994. doi: 10.1002/anie.202212994 (PMC9828546; doi:10.1002/anie.202212994)
Supplement: Supplementary file 1 — Supporting Information [file ANIE-61-0-s001.pdf]

Angewandte Chemie
Eine Zeitschrift der Gesellschaft Deutscher Chemiker

Supporting Information

FRET Monitoring of a Nonribosomal Peptide Synthetase Elongation Module Reveals Carrier Protein Shuttling between Catalytic Domains

*J. Rüschenbaum, W. Steinchen, F. Mayerthaler, A.-L. Feldberg, H. D. Mootz**

Experimental Section	page S2
Supplementary Tables	page S6
Supplementary Figures	page S11
Supporting References	page S26

EXPERIMENTAL SECTION

General information

ATP, coenzyme A, DTT, HEPES, IPTG and $MgCl_2$ were purchased from AppliChem. L-Pro, PP_i and TCEP were purchased from Carl Roth. L-Phe, inorganic pyrophosphatase, AF555 and AF647 maleimides were purchased from Thermo Fisher Scientific. AMP and desulfo-coenzyme A were purchased from Jena Bioscience. Ni-NTA agarose was purchased from Cube Biotech GmbH. Oligonucleotides were purchased from Biolegio. The TycB1 cysteine free sequence was ordered as a GeneArt™ String™ DNA Fragment from Invitrogen. Other chemicals were purchased from AppliChem, Carl Roth or Thermo Fisher Scientific. Experimental data were analyzed and fitted using GraphPad Prism 3.

Cloning of expression plasmids

The sequence encoding the cysteine free TycB1 module was ordered as a GeneArt™ String™ DNA Fragment with restriction sites NcoI and SmaI on the 5'- and 3'-ends, respectively. The DNA Fragment and backbone plasmid (pTrc99a vector, including His_6-tag) were digested with restriction enzymes NcoI and SmaI, gel extracted and ligated (pJR29, [TycB1,Δ] C-A-PCP-His_6). For insertion of C657 and C662 plasmid pJR29 was mutated using primers oJR176 (5'-CTGCAGTATACCACCTGCAGCTTTGATGTTTGCTATCAAGAAATCTTTTC-3') and oJR177 (5'-GAAAAGATTTCTTGATAGCAAACATCAAAGCTGCAGGTGGTATACTGCAG-3') to give plasmid pJR117. The GVCTE-tag was introduced by using primers oJR168 (5'-GGAAGGATCCAGATCTGGCGTGTGCACCGAACATCACCATCACCATC-3') and oJR169 (5'-GATGGTGATGGTGATGTTCGGTGCACACGCCAGATCTGGATCCTTCC-3') to give plasmid pJR118 coding for [TycB1,Δ] C-A(C657,C662)-PCP-GVCTE-His_6. Plasmid pJR118 was used as a template to produce the plasmids coding for the full-length sensors by insertion of mutation A553C (oJR178 (5'-GATCCGGAACTGCCTTGTGAACGTATTGCCTATATGC-3') and oJR179 (5'-GCATATAGGCAATACGTTCACAAGGCAGTTCCGGATC-3')) to give pJR120 or E191C (oJR138 (5'-CGTTTTATCAATTGGCTGTGCAAACAGAATAAACAGGC-3') and oJR139 (5'-GCCTGTTTATTCTGTTTGCACAGCCAATTGATAAAACG-3')) to give pJR121 encoding [TycB1, Δ] C-A(A553C,C657,C662)-PCP-GVCTE-His_6 and [TycB1, Δ] C(E191C)-A(C657,C662)-PCP-GVCTE-His_6, respectively. pJR120 and pJR121 were used as templates to introduce mutation H35tag using primers oJR212 (5'-GCTGGATCAAGAACATAATAGCTAGCTGGTTCAGATGAGC-3') and oJR213 (5'-GCTCATCTGAACCAGCTAGCTATTATGTTCTTGATCCAGC-3') to yield plasmids pJR139 ([TycB1, Δ] C(H35tag)-A(A553C,C657,C662)-PCP-GVCTE-His_6) and pJR141 ([TycB1, Δ] C(H35tag,E191C)-A(C657,C662)-PCP-GVCTE-His_6) for nonsense suppression. pJR120 and pJR121 were used as templates to introduce mutation Y337tag using primers oJR216 (5'-GTATGAACATGTGCCGCTGTAGGATATTCAGACACAGAGC-3') and oJR217 (5'-GCTCTGTGTCTGAATATCCTACAGCGGCACATGTTCATAC-3') to give plasmids pJR142 ([TycB1, Δ] C(Y337tag)-A(A553C,C657,C662)-PCP-GVCTE-His_6) and pJR144 ([TycB1, Δ] C(E191C,Y337tag)-A(C657,C662)-PCP-GVCTE-His_6). The plasmids pJR120 and pJR121 were used as templates to introduce mutation H35Y using primers oJR246

(5’-CAAGAACATAATAGCTATCTGGTTCAGATGAGC-3’) and oJR247 (5’-GCTCATCTGAACCAGATAGCTATTATGTTCTTG-3’) to give plasmids pJR162 and pJR163 encoding [TycB1, Δ] C(H35Y)-A(A553C,C657,C662)-PCP-GVCTE-His_6) and ([TycB1, Δ] C(E191C,Y337Y)-A(C657,C662)-PCP-GVCTE-His_6),respectively. Primers oJR194 (5’-GCAGTTGCAAATAATACCATGGTTGAACTGGCACAG-3‘) and oJR195 (5’-CTGTGCCAGTTCAACCATGGTATTATTTGCAACTGC-3‘) were used to introduce restriction site NcoI in plasmid pJR120 in the sequence linker region between the C-domain and A-domain. The resulting plasmid was digested with NcoI, the fragment gel extracted and religated resulting in deletion of C-domain sequence to yield plasmid pJR130 encoding [TycB1, Δ] A(A553C,C657,C662)-PCP-GVCTE-His_6. To generate a [GrsA] A-PCP sensor with similar design as the TycB1 sensors, pAF17 ([GrsA, Δ] A-PCP-RSEGVCTE-His_6 was used as template to introduce mutation K125C using primers oJR210 (5’-GGTGGAGCATATGTTCCGATTGATATTGAATATCCTTGTGAAAGAATTCAATATATTC-3’) and oJR211 (5’-GAATATATTGAATTCTTTCACAAGGATATTCAATATCAATCGGAACATATGCTCCACC-3’) to give plasmid pJR138 encoding [GrsA, Δ] A(K125C)-PCP-RSEGVCTE-His_6.

Gene overexpression and protein purification

Genes were expressed and purified as previously described,[1] using the *E.coli* EC100 (ΔEntD) strain. After purification and dialysis, the concentration of all constructs was determined through their absorption at 280 nm, using the theoretical extinction coefficient calculated by ProtParam.[2] The unnatural amino acid ONBY was incorporated using nonsense suppression.[3] The corresponding pEVOL plasmid was introduced together with the encoding plasmid into the *E.coli* expression strain. ONBY was added in a final concentration of 1 mM to the growth medium.

Chemical modification with AF555 and AF647 maleimides

Proteins (4 µM) in NRPS assay buffer (50 mM HEPES, 100 mM NaCl, 1 mM EDTA, 10 mM $MgCl_2$) were incubated with 2 mM TCEP for 30 min at 18°C to reduce cysteine side chains. AF555 and AF647 maleimides stock solutions in DMSO (10 µM) were premixed at a 7 to 8 equivalent ratio and added directly to the protein solution. The labeling reaction was performed for 30 min at 18°C and quenched by addition of 2 mM DTT. The labeled proteins were added to a Ni-NTA column (200 µL column material), washed with 2 mL NRPS assay buffer, and afterwards eluted with NRPS assay buffer containing 250 mM imidazole. Fractions containing the labeled protein of interest were combined, post-translationally converted overnight on ice in the active form and then dialyzed in NRPS assay buffer.

Post-translational conversion of apo into holo proteins

Apo proteins were incubated in NRPS assay buffer with 2 mM TCEP, 10 mM $MgCl_2$, 50 equivalents coenzyme A or desulfo coenzyme A (25 equivalents) and 0.02 equivalents phosphopantetheine transferase Sfp from *Bacillus subtilis*. The reaction mixture was incubated

on ice overnight, followed by removal of excess CoA, TCEP and $MgCl_2$ by dialysis against NRPS assay buffer.

MS assay to detect thioester formation

The formation of the proline thioester by the unlabeled and labeled enzymes was followed by ESI-TOF-MS. For the reaction the enzyme (10 µM) in NRPS assay buffer was incubated at 25°C with 2 mM each of ATP and L-proline. At respective time points the reaction was quenched by adding 45 µL of reaction mixture to 5 µL of a 10% formic acid solution. Samples were centrifuged (10 min, 15000 rpm, 4°C), 30 µL were transferred to mass vials and directly measured to prohibit precipitation. The mass analysis was performed as described earlier.[4]

FRET measurements

The measurements were basically performed as previously outlined.[4] FRET measurements were performed using either a 384-well plate (Greiner) together with the Tecan INFINITE M1000 Pro Microplate Reader (Tecan Austria GmbH) or a quartz cuvette (Hellma) together with the FP-8300 Fluorescence Spectrometer (Jasco Deutschland GmbH). As direct excitation wavelength 520 nm was chosen for AF555 maleimide and 650 nm for AF647 maleimide. The respective emission wavelengths were detected at 570 nm for AF555 and 674 nm for AF647. For INFINITE M1000 Pro and for FP-8300 a monochromator for both excitation as well as emission with a bandwidth of 5 nm was applied. With this setup for each measurement the fluorescence of both AF555 and AF647 after direct excitation was recorded as well as the emission of AF647 after AF555 excitation (energy transfer). For endpoint measurements (INFINITE M1000 Pro) data were taken after 30 min incubation with substrates or ligands, whereas for time-resolved measurements (FP-8300) readings were taken every minute. The sensors were used at a concentration of 300 nM (based on the acceptor fluorophore concentration) in a total volume of 50 µL assay buffer (pH 7) and incubated for 5 to 10 min until a stable fluorescence signal was recorded. Subsequently substrates or ligands were added as indicated in the figures (ATP and L-Pro at 2 mM, PP_i at 10 mM, PPase at 0.2 U). The obtained raw data were normalized using the emission of AF647 after its direct excitation to eliminate fluorophore fluctuations based on varying enzyme concentrations. Additionally, the recorded energy transfer was corrected for the donor bleed-through and the crosstalk. The bleed-through was treated as a fraction of AF555 emission at 674 nm (6.66% for INFINITE M1000 Pro and 0.22% for FP-8300). The crosstalk designates the direct excitation of AF647 at 520 nm, calculated by excitation of the standalone dye, and was determined as a fraction of the observed fluorescence after excitation (5.38% for INFINITE M1000 Pro) and 1.41% for FP-8300).[5] The FRET ratio was calculated of the corrected data and normalized to 1 as the time point before substrate addition. Each experiment was performed with three biological replicates each containing multiple technical repeats. The mean and SD of each data sets were calculated and clustered. The combined SDs (S_C) were calculated using

$$S_C{}^2 = \frac{\sum_{i=1}^{k} n_i \, [S_i{}^2 + (X_i - X_C)^2]}{\sum_{i=1}^{k} n_i},$$

using X_i as mean of individual and X_C as means of combined data sets.

D-Phe-L-Pro-diketopiperazine (DKP) formation assay

The DKP formation assay was basically performed as previously described.[6] 5 µM of full-length holo-TycB1 was incubated with 5 mM ATP in assay buffer (pH 7.0). The reaction was started by adding 1 mM L-Pro, 1mM L-Phe, 10 mM $MgCl_2$ and 0.5 µM of the partner initiation module holo-GrsA out of a master mixture, incubated at 37°C and quenched after 30 min by addition of 400 µL n-butanol/chloroform (4:1 v/v) and 200 µL ddH_2O. The organic phase was extracted and the aqueous phase washed with 400 µL of the organic mixture. The organic phases were combined and afterwards washed twice with 300 µL ddH_2O. After each washing the solutions were vortexed for 20 sec and centrifuged (2 min, 13000 rpm, 25°C) to separate the phases. The resulting organic phase was dried under vacuum and the remaining resolved in 30 µL of HPLC starting conditions (95% ddH_2O, 5% acetonitrile and 0.1% trifluoroacetic acid). The solution was analyzed via HPLC (reversed-phase C18 column) and the area under the curve of the 210 nm UV/vis signal trail was used for the quantification of DKP formation.

Hydrogen-deuterium exchange mass spectrometry (HDX-MS)

50 µM holo wildtype TycB1 was incubated for 30 minutes at room temperature either without any ligand (setting i) or in presence of 1 mM of each ATP and L-Pro (setting ii). The following sample preparation and measurement was performed as previously reported.[4, 7]

Source data for HDX-MS experiments are contained in the Supplemental Dataset, which also contains the further analysis with respect to differences in sequences stretches (seq) between the settings i and ii of holo wildtype TycB1. In short, the difference in deuterium incorporation between setting ii and that of setting i was calculated for each peptide and HDX timepoint. From that, the sum of differences over all sampled timepoints was calculated. In order illustrate the data for all peptides (out of which there are multiple overlapping ones, see Fig. S9) in a single plot, a peptide midpoint was calculated for each by addition of the first and last residue constituting the peptide followed by division with 2. Plotting the sum of the differences versus the peptide midpoints yields Fig. S11.[8] In order to define the boundaries between a peptide exhibiting no difference in HDX and one that shows HDX reduction, the peptide midpoints of these peptides were summed and divided by 2; a similar procedure was applied to define the boundaries between peptides exhibiting HDX differences of dissimilar amplitude (compare to Fig. 4A). This procedure results in sequence stretches (seq) that may differ in length from the length of the peptides, i.e., seq's may be longer than individual peptides in cases where multiple consecutive peptides exhibit a significant difference in HDX between settings ii and i (for example seq16, Fig. S10), or seq's may be shorter than individual peptides in cases where overlapping peptides were differently affected in HDX of setting ii versus i (for example seq5).

SUPPLEMENTARY TABLES

Table S1. List of recombinantly produced proteins used in this study and their encoding plasmids, all generated in this study except pGV196.[6b]

**Name of construct***	**Encoding plasmid**	**Vector backbone**
[TycB1] C-A-PCP- His_6	pJR95 WT	pTrc99a
[TycB1, Δ] C-A(C657,C662)-PCP-His_6	pJR117	pTrc99a
[TycB1, Δ] C-A(A553C,C657,C662)-PCP-GVCTE-His_6 > C-$A^{\#}$-$PCP^{\#}$	pJR120	pTrc99a
[TycB1, Δ] C(E191C)-A(C657,C662)-PCP-GVCTE-His_6 > $C^{\#}$-A-$PCP^{\#}$	pJR121	pTrc99a
[TycB1, Δ] A(A553C,C657,C662)-PCP-GVCTE-His_6 > $A^{\#}$-$PCP^{\#}$	pJR130	pTrc99a
[TycB1, Δ] C(H35X)-A(A553C,C657,C662)-PCP-GVCTE-His_6 > C(H35X)-$A^{\#}$-$PCP^{\#}$	pJR139	pTrc99a
[TycB1, Δ] C(Y337X)-A(A553C,C657,C662)-PCP-GVCTE-His_6 > C(Y337X)-$A^{\#}$-$PCP^{\#}$	pJR141	pTrc99a
[TycB1, Δ] C(H35X,E191C)-A(C657,C662)-PCP-GVCTE-His_6 > $C^{\#}$(H35X)-A-$PCP^{\#}$	pJR142	pTrc99a
[TycB1, Δ] C(E191C,Y337X)-A(C657,C662)-PCP-GVCTE-His_6 > $C^{\#}$(Y337X)-A-$PCP^{\#}$	pJR144	pTrc99a
[TycB1, Δ] C(H35Y)-A(A553C,C657,C662)-PCP-GVCTE-His_6 > C(H35Y)-$A^{\#}$-$PCP^{\#}$	pJR162	pTrc99a
[TycB1, Δ] C(H35Y,E191C)-A(C657,C662)-PCP-GVCTE-His_6 > $C^{\#}$(H35Y)-A-$PCP^{\#}$	pJR163	pTrc99a
[GrsA] SBP-A-PCP-E- His_6	pGV196	pET28a
[GrsA, Δ] A(K125C)-PCP-RSEGVCTE-His_6	pJR138	pET28a

* X denotes amino acid incorporation in response to amber stop codon.

Table S2. Sequences of recombinantly produced proteins

Protein	Sequence
[GrsA] SBP-A-PCP-E-His$_6$	MDEKTTGWRGGHVVEGLAGELEQLRARLEHHPQGQREPGASMLNSSKSILIHA<br>QNKNGTHEEEQYLFAVNNTKAEYPRDKTIHQLFEEQVSKRPNNVAIVCENEQLT<br>YHELNVKANQLARIFIEKGIGKDTLVGIMMEKSIDLFIGILAVLKAGGAYVPIDIE<br>YPKERIQYILDDSQARMLLTQKHLVHLIHNIQFNGQVEIFEEDTIKIREGTNLHVP<br>SKSTDLAYVIYTSGTTGNPKGTMLEHKGISNLKVFFENSLNVTEKDRIGQFASISF<br>DASVWEMFMALLTGASLYIILKDTINDFVKFEQYINQKEITVITLPPTYVVHLDPE<br>RILSIQTLITAGSATSPSLVNKWKEKVTYINAYGPTETTICATTWVATKETIGHSV<br>PIGAPIQNTQIYIVDENLQLKSVGEAGELCIGGEGLARGYWKRPELTSQKFVDNP<br>FVPGEKLYKTGDQARWLSDGNIEYLGRIDNQVKIRGHRVELEEVESILLKHMYIS<br>ETAVSVHKDHQEQPYLCAYFVSEKHIPLEQLRQFSSEELPTYMIPSYFIQLDKMPL<br>TSNGKIDRKQLPEPDLTFGMRVDYEAPRNEIEETLVTIWQDVLGIEKIGIKDNFYA<br>LGGDSIKAIQVAARLHSYQLKLETKDLLKYPTIDQLVHYIKDSKRRSEQGIVEGEI<br>GLTPIQHWFFEQQFTNMHHWNQSYMLYRPNGFDKEILLRVFNKIVEHHDALRMI<br>YKHHNGKIVQINRGLEGTLFDFYTFDLTANDNEQQVICEESARLQNSINLEVGPL<br>VKIALFHTQNGDHLFMAIHHLVVDGISWRILFEDLATAYEQAMHQQTIALPEKT<br>DSFKDWSIELEKYANSELFLEEAEYWHHLNYYTENVQIKKDYVTMNNKQKNIR<br>YVGMELTIEETEKLLKNVNKAYRTEINDILLTALGFALKEWADIDKIVINLEGHG<br>REEILEQMNIARTVGWFTSQYPVVLDMQKSDDLSYQIKLMKENLRRIPNKGIGY<br>EIFKYLTTEYLRPVLPFTLKPEINFNYLGQFDTDVKTELFTRSPYSMGNSLGPDGK<br>NNLSPEGESYFVLNINGFIEEGKLHITFSYNEQQYKEDTIQQLSRSYKQHLLAIIEH<br>CVQKEDTELTPSDFSFKELELEEMDDIFDLLADSLTGSRSHHHHHH
[TycB1] C-A-PCP-His$_6$	MSVFSKEQVQDMYALTPMQEGMLFHALLDQEHNSHLVQMSISLQGDLDVGLFT<br>DSLHVLVERYDVFRTLFLYEKLKQPLQVVLKQRPIPIEFYGLSACDESEKQLRYT<br>QYKRADQERTFHLAKDPLMRVALFQMSQHDYQVIWSFHHILMDGWCFSIIFDD<br>LLAIYLSLQNKTALSLEPVQPYSRFINWLEKQNKQAALNYWSDYLEAYEQKTTL<br>PKKEAAFAKAFQPTQYRFSLNRTLTKQLGTIASQNQVTLSTVIQTIWGVLLQKYN<br>AAHDVLFGSVVSGRPTDIVGIDKMVGLFINTIPFRVQAKAGQTFSELLQAVHKRT<br>LQSQPYEHVPLYDIQTQSVLKQELIDHLLVIENYPLVEALQKKALNQQIGFTITAV<br>EMFEPTNYDLTVMVMPKEELAFRFDYNAALFDEQVVQKLAGHLQQIADCVAN<br>NSGVELCQIPLLTEAETSQLLAKRTETAADYPAATMHELFSRQAEKTPEQVAVV<br>FADQHLTYRELDEKSNQLARFLRKKGIGTGSLVGTLLDRSLDMIVGILGVLKAG<br>GAFVPIDPELPAERIAYMLTHSRVPLVVTQNHLRAKVTTPTETIDINTAVIGEESR<br>APIESLNQPHDLFYIIYTSGTTGQPKGVMLEHRNMANLMHFTFDQTNIAFHEKVL<br>QYTTCSFDVCYQEIFSTLLSGGQLYLITNELRRHVEKLFAFIQEKQISILSLPVSFL<br>KFIFNEQDYAQSFPRCVKHIITAGEQLVVTHELQKYLRQHRVFLHNHYGPSETHV<br>VTTCTMDPGQAIPELPPIGKPISNTGIYILDEGLQLKPEGIVGELYISGANVGRGYL<br>HQPELTAEKFLDNPYQPGERMYRTGDLARWLPDGQLEFLGRIDHQVKIRGHRIE<br>LGEIESRLLNHPAIKEAVVIDRADETGGKFLCAYVVLQKALSDEEMRAYLAQAL<br>PEYMIPSFFVTLERIPVTPNGKTDRRALPKPEGSAKTKADYVAPTTELEQKLVAI<br>WEQILGVSPIGIQDHFFTLGGHSLKAIQLISRIQKECQADVPLRVLFEQPTIQALAA<br>YVEGSRSHHHHHH
[TycB1, Δ] C-A(A553C)-PCP(1051C)-His$_6$	MGVFSKEQVQDMYALTPMQEGMLFHALLDQEHNSHLVQMSISLQGDLDVGLF<br>TDSLHVLVERYDVFRTLFLYEKLKQPLQVVLKQRPIPIEFYDLSAADESEKQLRY<br>TQYKRADQERTFHLAKDPLMRVALFQMSQHDYQVIWSFHHILMDGWSFSIIFDD<br>LLAIYLSLQNKTALSLEPVQPYSRFINWLEKQNKQAALNYWSDYLEAYEQKTTL<br>PKKEAAFAKAFQPTQYRFSLNRTLTKQLGTIASQNQVTLSTVIQTIWGVLLQKYN<br>AAHDVLFGSVVSGRPTDIVGIDKMVGLFINTIPFRVQAKAGQTFSELLQAVHKRT<br>LQSQPYEHVPLYDIQTQSVLKQELIDHLLVIENYPLVEALQKKALNQQIGFTITAV<br>EMFEPTNYDLTVMVMPKEELAFRFDYNAALFDEQVVQKLAGHLQQIADAVAN<br>NSGVELAQIPLLTEAETSQLLAKRTETAADYPAATMHELFSRQAEKTPEQVAVV<br>FADQHLTYRELDEKSNQLARFLRKKGIGTGSLVGTLLDRSLDMIVGILGVLKAG<br>GAFVPIDPELPAERIAYMLTHSRVPLVVTQNHLRAKVTTPTETIDINTAVIGEESR<br>APIESLNQPHDLFYIIYTSGTTGQPKGVMLEHRNMANLMHFTFDQTNIAFHEKVL<br>QYTTCSFDVCYQEIFSTLLSGGQLYLITNELRRHVEKLFAFIQEKQISILSLPVSFL<br>KFIFNEQDYAQSFPRSVKHIITAGEQLVVTHELQKYLRQHRVFLHNHYGPSETHV<br>VTTATMDPGQAIPELPPIGKPISNTGIYILDEGLQLKPEGIVGELYISGANVGRGYL<br>HQPELTAEKFLDNPYQPGERMYRTGDLARWLPDGQLEFLGRIDHQVKIRGHRIE<br>LGEIESRLLNHPAIKEAVVIDRADETGGKFLAAYVVLQKALSDEEMRAYLAQAL<br>PEYMIPSFFVTLERIPVTPNGKTDRRALPKPEGSAKTKADYVAPTTELEQKLVAI<br>WEQILGVSPIGIQDHFFTLGGHSLKAIQLISRIQKEAQADVPLRVLFEQPTIQALAA<br>YVEGSRSGVCTEHHHHHH

[TycB1, Δ] A(A553C)-PCP(1051C)-His_6	MVELAQIPLLTEAETSQLLAKRTETAADYPAATMHELFSRQAEKTPEQVAVVFA<br>DQHLTYRELDEKSNQLARFLRKKGIGTGSLVGTLLDRSLDMIVGILGVLKAGGA<br>FVPIDPELPCERIAYMLTHSRVPLVVTQNHLRAKVTTPTETIDINTAVIGEESRAPI<br>ESLNQPHDLFYIIYTSGTTGQPKGVMLEHRNMANLMHFTFDQTNIAFHEKVLQY<br>TTCSFDVCYQEIFSTLLSGGQLYLITNELRRHVEKLFAFIQEKQISILSLPVSFLKFI<br>FNEQDYAQSFPRSVKHIITAGEQLVVTHELQKYLRQHRVFLHNHYGPSETHVVT<br>TATMDPGQAIPELPPIGKPISNTGIYILDEGLQLKPEGIVGELYISGANVGRGYLHQ<br>PELTAEKFLDNPYQPGERMYRTGDLARWLPDGQLEFLGRIDHQVKIRGHRIELG<br>EIESRLLNHPAIKEAVVIDRADETGGKFLAAYVVLQKALSDEEMRAYLAQALPE<br>YMIPSFFVTLERIPVTPNGKTDRRALPKPEGSAKTKADYVAPTTELEQKLVAIWE<br>QILGVSPIGIQDHFFTLGGHSLKAIQLISRIQKEAQADVPLRVLFEQPTIQALAAYV<br>EGSRSGVCTEHHHHHH
[TycB1, Δ] C(E191C)-A-PCP(1051C)-His_6	MGVFSKEQVQDMYALTPMQEGMLFHALLDQEHNSHLVQMSISLQGDLDVGLF<br>TDSLHVLVERYDVFRTLFLYEKLKQPLQVVLKQRPIPIEFYDLSAADESEKQLRY<br>TQYKRADQERTFHLAKDPLMRVALFQMSQHDYQVIWSFHHILMDGWSFSIIFDD<br>LLAIYLSLQNKTALSLEPVQPYSRFINWLCKQNKQAALNYWSDYLEAYEQKTTL<br>PKKEAAFAKAFQPTQYRFSLNRTLTKQLGTIASQNQVTLSTVIQTIWGVLLQKYN<br>AAHDVLFGSVVSGRPTDIVGIDKMVGLFINTIPFRVQAKAGQTFSELLQAVHKRT<br>LQSQPYEHVPLYDIQTQSVLKQELIDHLLVIENYPLVEALQKKALNQQIGFTITAV<br>EMFEPTNYDLTVMVMPKEELAFRFDYNAALFDEQVVQKLAGHLQQIADAVAN<br>NSGVELAQIPLLTEAETSQLLAKRTETAADYPAATMHELFSRQAEKTPEQVAVV<br>FADQHLTYRELDEKSNQLARFLRKKGIGTGSLVGTLLDRSLDMIVGILGVLKAG<br>GAFVPIDPELPAERIAYMLTHSRVPLVVTQNHLRAKVTTPTETIDINTAVIGEESR<br>APIESLNQPHDLFYIIYTSGTTGQPKGVMLEHRNMANLMHFTFDQTNIAFHEKVL<br>QYTTCSFDVCYQEIFSTLLSGGQLYLITNELRRHVEKLFAFIQEKQISILSLPVSFL<br>KFIFNEQDYAQSFPRSVKHIITAGEQLVVTHELQKYLRQHRVFLHNHYGPSETHV<br>VTTATMDPGQAIPELPPIGKPISNTGIYILDEGLQLKPEGIVGELYISGANVGRGYL<br>HQPELTAEKFLDNPYQPGERMYRTGDLARWLPDGQLEFLGRIDHQVKIRGHRIE<br>LGEIESRLLNHPAIKEAVVIDRADETGGKFLAAYVVLQKALSDEEMRAYLAQAL<br>PEYMIPSFFVTLERIPVTPNGKTDRRALPKPEGSAKTKADYVAPTTELEQKLVAI<br>WEQILGVSPIGIQDHFFTLGGHSLKAIQLISRIQKEAQADVPLRVLFEQPTIQALAA<br>YVEGSRSGVCTEHHHHHH
[GrsA, Δ] A(K125C)-PCP(616C) -His_6	MVNSSKSILIHAQNKNGTHEEEQYLFAVNNTKAEYPRDKTIHQLFEEQVSKRPN<br>NVAIVFENEQLTYHELNVKANQLARIFIEKGIGKDTLVGIMMEKSIDLFIGILAVL<br>KAGGAYVPIDIEYPCERIQYILDDSQARMLLTQKHLVHLIHNIQFNGQVEIFEEDT<br>IKIREGTNLHVPSKSTDLAYVIYTSGTTGNPKGTMLEHKGISNLKVFFENSLNVTE<br>KDRIGQFASISFDASVWEMFMALLTGASLYIILKDTINDFVKFEQYINQKEITVITL<br>PPTYVVHLDPERILSIQTLITAGSATSPSLVNKWKEKVTYINAYGPTETTIAATTW<br>VATKETIGHSVPIGAPIQNTQIYIVDENLQLKSVGEAGELSIGGEGLARGYWKRPE<br>LTSQKFVDNPFVPGEKLYKTGDQARWLSDGNIEYLGRIDNQVKIRGHRVELEEV<br>ESILLKHMYISETAVSVHKDHQEQPYLAAYFVSEKHIPLEQLRQFSSEELPTYMIP<br>SYFIQLDKMPLTSNGKIDRKQLPEPDLTFGMRVDYEAPRNEIEETLVTIWQDVLG<br>IEKIGIKDNFYALGGDSIKAIQVAARLHSYQLKLETKDLLKYPTIDQLVHYIKDSK<br>RGVCTEHHHHHH

Δ represents following mutations: Cys removed (C98A, C154S, C430A, C440A, C725S, C768A, C907A, C1021A), Cys remaining (C657, C662).

Table S3. List of designed constructs with informations about sequence, posttranslational modifications and application.

No.	parent modul	domain composition	sequence information	Fluorophore bioconjugation	ppantylation	Calculated mass (Da)	Comment
**1**	TycB1	apo C-A-PCP	wt	No	No	119696.2	
**2**	TycB1	holo C-A-PCP	wt	No	Yes (Ppant)	120037.5	
**3**	TycB1	apo C-A-PCP	C98A,C154S,C430A,C440A, C725S,C768A,C907A, C1021A	No	No	119427.6	Construct generated for specific cysteine insertion
**4**	TycB1	holo C-A-PCP	C98A,C154S,C430A,C440A, C725S,C768A,C907A, C1021A	No	Yes (Ppant)	119769.0	
**5**	TycB1	apo C-A$^{\#}$-PCP$^{\#}$	A553C, 1051C	No	No	119949.2	
**6**	TycB1	holo C-A$^{\#}$-PCP$^{\#}$	A553C, 1051C	No	Yes (Ppant)	120290.6	
**7**	TycB1	apo C-A*- PCP*	A553C, 1051C	AF555+AF647	No	121885.3	
**8**	TycB1	holo C-A*-PCP*	A553C, 1051C	AF555+AF647	Yes (Ppant)	122226.7	FRET sensor for transfer conformation of the PCP-A interaction
**9**	TycB1	desulfo C-A*-PCP*	A553C, 1051C	AF555+AF647	Yes (desulfo-Ppant)	122194.6	FRET sensor for transfer conformation of the PCP-A interaction
**10**	TycB1	holo C-A$^{\#}$-PCP$^{\#}$	H35Y, A553C, 1051C	No	Yes (Ppant)	120316.6	
**11**	TycB1	apo C-A*- PCP*	H35ONBY, A553C, 1051C	AF555+AF647	No	120110.3	
**12**	TycB1	holo C-A*- PCP*	H35ONBY, A553C, 1051C	AF555+AF647	Yes (Ppant)	120451.7	FRET sensor for transfer conformation of the PCP-A interaction with obstructed acceptor site of C domain for PCP domain entry
**13**	TycB1	apo C-A*- PCP*	Y337ONBY, A553C, 1051C	AF555+AF647	No	120084.2	
**14**	TycB1	holo C-A*- PCP*	Y337ONBY, A553C, 1051C	AF555+AF647	Yes (Ppant)	120425.6	FRET sensor for transfer conformation of the PCP-A interaction with obstructed acceptor site of C domain for PCP domain entry
**15**	TycB1	apo C$^{\#}$-A-PCP$^{\#}$	E191C, 1051C	No	No	119891.2	
**16**	TycB1	holo C$^{\#}$-A-PCP$^{\#}$	E191C, 1051C	No	Yes (Ppant)	120232.5	
**17**	TycB1	apo C*-A- PCP*	E191C, 1051C	AF555+AF647	No	121827.3	
**18**	TycB1	holo C*-A- PCP*	E191C, 1051C	AF555+AF647	Yes (Ppant)	122168.6	FRET sensor for C conformation of the PCP-C interaction
**19**	TycB1	desulfo C*-A-PCP*	E191C, 1051C	AF555+AF647	Yes (desulfo-Ppant)	122136.6	FRET sensor for C conformation of the PCP-C interaction
**20**	TycB1	holo C$^{\#}$-A-PCP$^{\#}$	H35Y, E191C, 1051C	No	Yes (Ppant)	120258.6	Control construct for H35ONBY mutation (constructs **19** + **20**)
**21**	TycB1	apo C*-A-PCP*	H35ONBY, E191C, 1051C	AF555+AF647	No	120052.2	
**22**	TycB1	holo C*-A-PCP*	H35ONBY, E191C, 1051C	AF555+AF647	Yes (Ppant)	120393.6	FRET sensor for C conformation of the PCP-C interaction with obstructed acceptor site of C domain for PCP domain entry
**23**	TycB1	apo C*-A-PCP*	E191C,Y337ONBY, 1051C	AF555+AF647	No	120026.2	

**24**	TycB1	holo C*-A-PCP*	E191C,Y337ONBY, 1051C	AF555+AF647	Yes (Ppant)	120367.6	FRET sensor for C conformation of the PCP-C interaction with obstructed acceptor site of C domain for PCP domain entry
**25**	TycB1	apo A#-PCP#	A553C, 1051C	No	No	69988.5	
**26**	TycB1	holo A#-PCP#	A553C, 1051C	No	Yes (Ppant)	70329.9	
**27**	TycB1	apo A*- PCP*	A553C, 1051C	AF555+AF647	No	71834.6	
**28**	TycB1	holo A*-PCP*	A553C, 1051C	AF555+AF647	Yes (Ppant)	72175.9	FRET sensor for transfer conformation of the PCP-A interaction
**29**	TycB1	desulfo A*- PCP*	A553C, 1051C	AF555+AF647	Yes (desulfo-Ppant)	72143.9	FRET sensor for transfer conformation of the PCP-A interaction
**30**	GrsA	holo A-PCP-E	wt	No	Yes (Ppant)	132685.4	
**31**	GrsA	holo A*-PCP*	K125C,616C	AF555+AF647	Yes (Ppant)	71279.6	FRET sensor for transfer conformation of the PCP-A interaction

Contructs **3** to **29** include following sequence modifications compared **1** or **2**: Cys removed (C98A,C154S,C430A,C440A,C725S,C768A,C907A, C1021A), Cys remaining (C657,C662); construct **31** includes following sequence modifications: Cys removed (C60F,C331A,C376A,C473A,C716S, C1063S)

SUPPLEMENTARY FIGURES

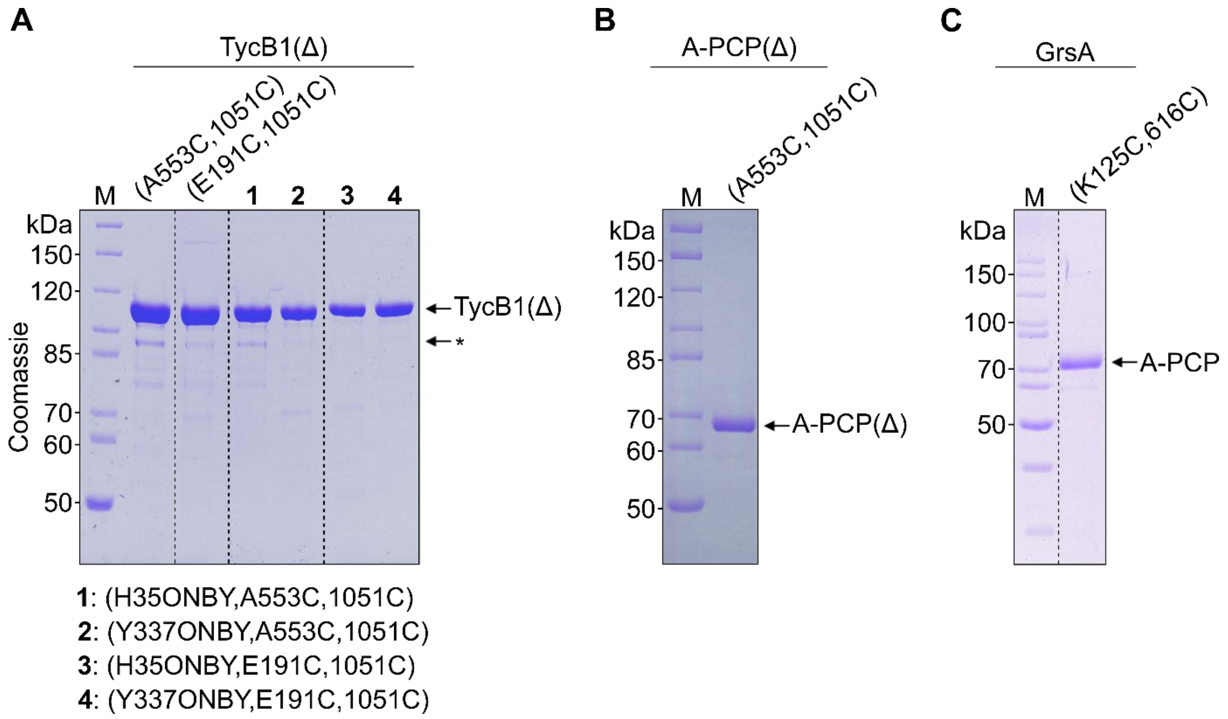

Figure S1. Purified proteins prior to fluorophore bioconjugation. Shown are the Coomassie-stained SDS-PAGE gels of (A) the unlabeled TycB1 sensors $C^{\#}$-A-$PCP^{\#}$ (E191C, 1051C), C-$A^{\#}$-$PCP^{\#}$ (A553C, 1051C), their ONBY mutants; (B) the TycB1 $A^{\#}$-$PCP^{\#}$ construct lacking the C domain; as well as (C) the new GrsA A-PCP didomain sensor. The positions 1051C and 616C represents a cysteine that was appended in a short sequence (GVCTE) to the C terminus of the PCP. The asterisk denotes minor protein contaminations.

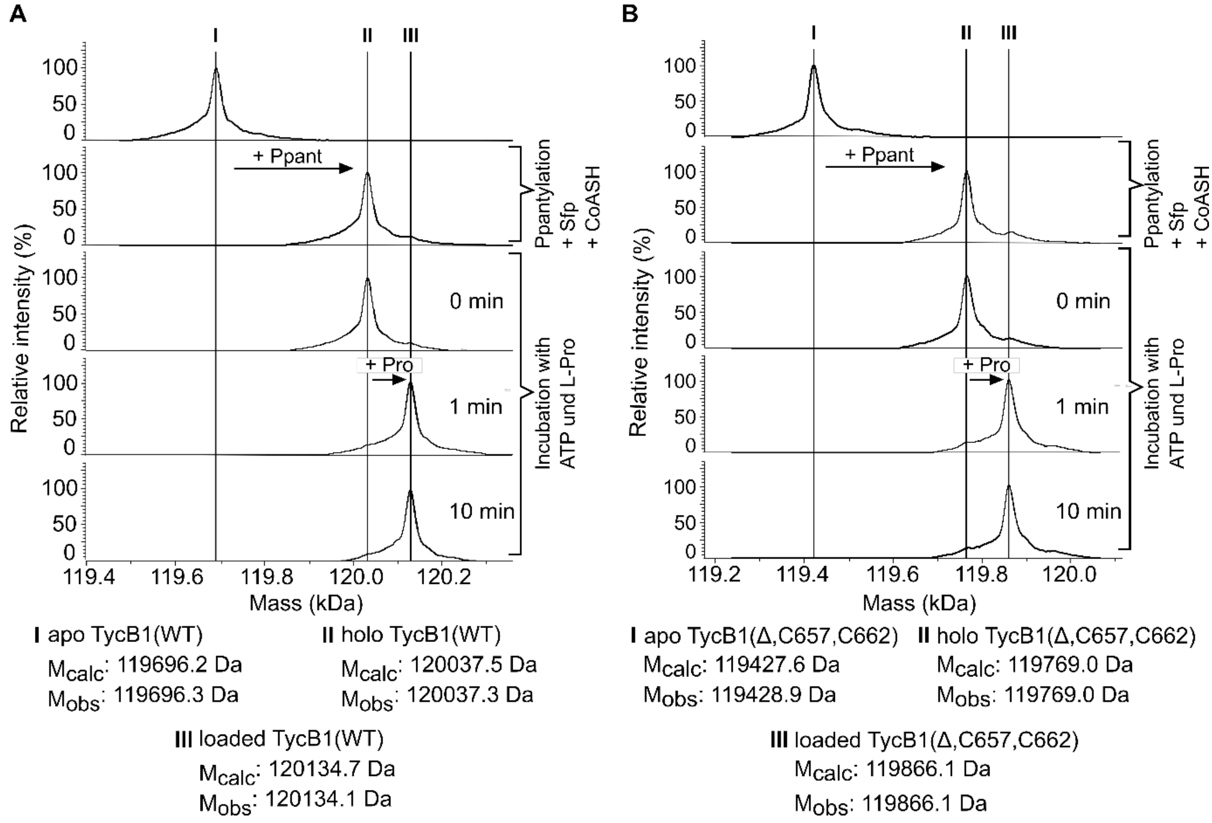

Figure S2. Tolerance to cysteine mutations in the establishment of TycB1 sensors. Shown are the MS analyses of Ppantylation and enzymatic activity in the aminoacylation reaction for the proteins: (A) TycB1 (wt) and (B) TycB1(ΔCys, C657, C662). TycB1(wt) is virtually fully loaded with its specific amino acid L-Pro after 1 min. By keeping the two cysteines located in the N-terminal subdomain of the A-domain in TycB1(ΔCys, C657, C662) the catalytic activity of the wildtype TycB1 module was retained and the protein was virtually completely charged after 1 min. Calculated and observed masses for apo, holo and loaded proteins are shown in the Figure.

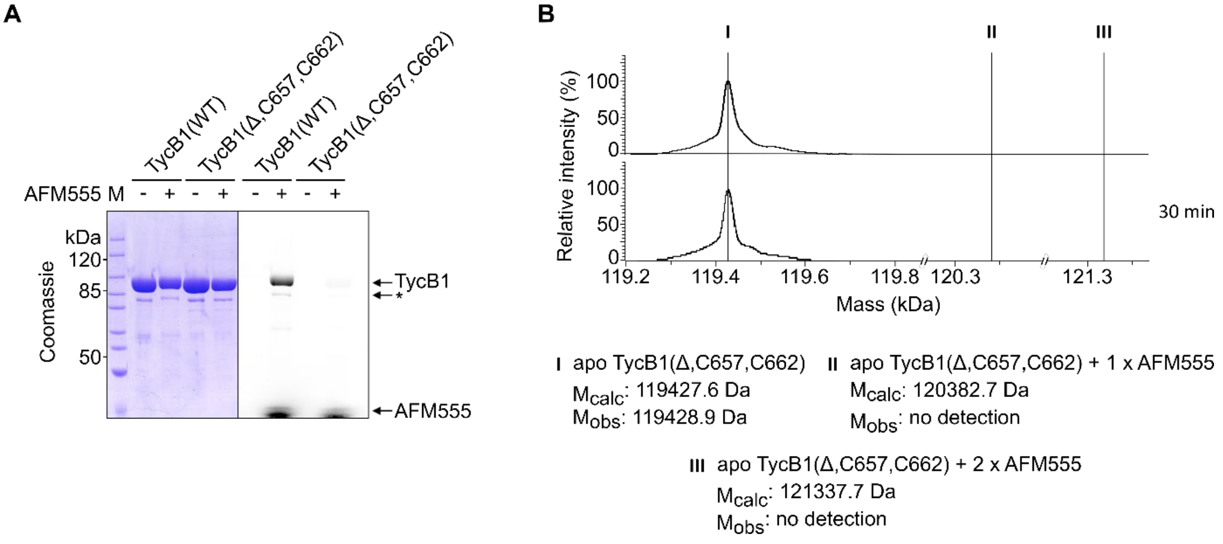

Figure S3. Control experiments to show Cys657 and Cys662 are not affected by maleimide bioconjugation. TycB1(wt) with all native cysteines and the TycB1 mutant TycB1(ΔCys, C657, C662) with only the two cysteines remaining were compared before and after treatment with Alexa Fluor® 555 maleimide (AF555). The proteins (at 4 µM each) were incubated with 10 Eq. of TCEP for 30 min at 18°C to reduce all cysteines. Then 5 Eq. of AF555 were added for 30 min at 18°C and finally the reaction was quenched by addition of 2 mM DTT. These steps were performed in HEPES buffer (50 mM HEPES, 100 mM NaCl, 10 mM $MgCl_2$, 1 mM EDTA) at pH 7.0 (A) Shown is an SDS-PAGE gel analysis using a fluorescent read-out (right panel) and Coomassie-staining (left panel). (B) Analysis of TycB1(ΔCys, C657, C662) by ESI-MS. Calculated masses are 119.8 kDa (TycB1(WT)), 119.4 kDa (TycB1(Δ, C657, C662)), 120.4 kDa (TycB1(Δ, C657, C662) + 1 x AF555 (955.04 Da) and 121.3 kDa (TycB1(Δ, C657, C662) + 2 x AF555). Together, these results show that Cys657 and Cys662 were unreactive for fluorophore bioconjugation under these conditions.

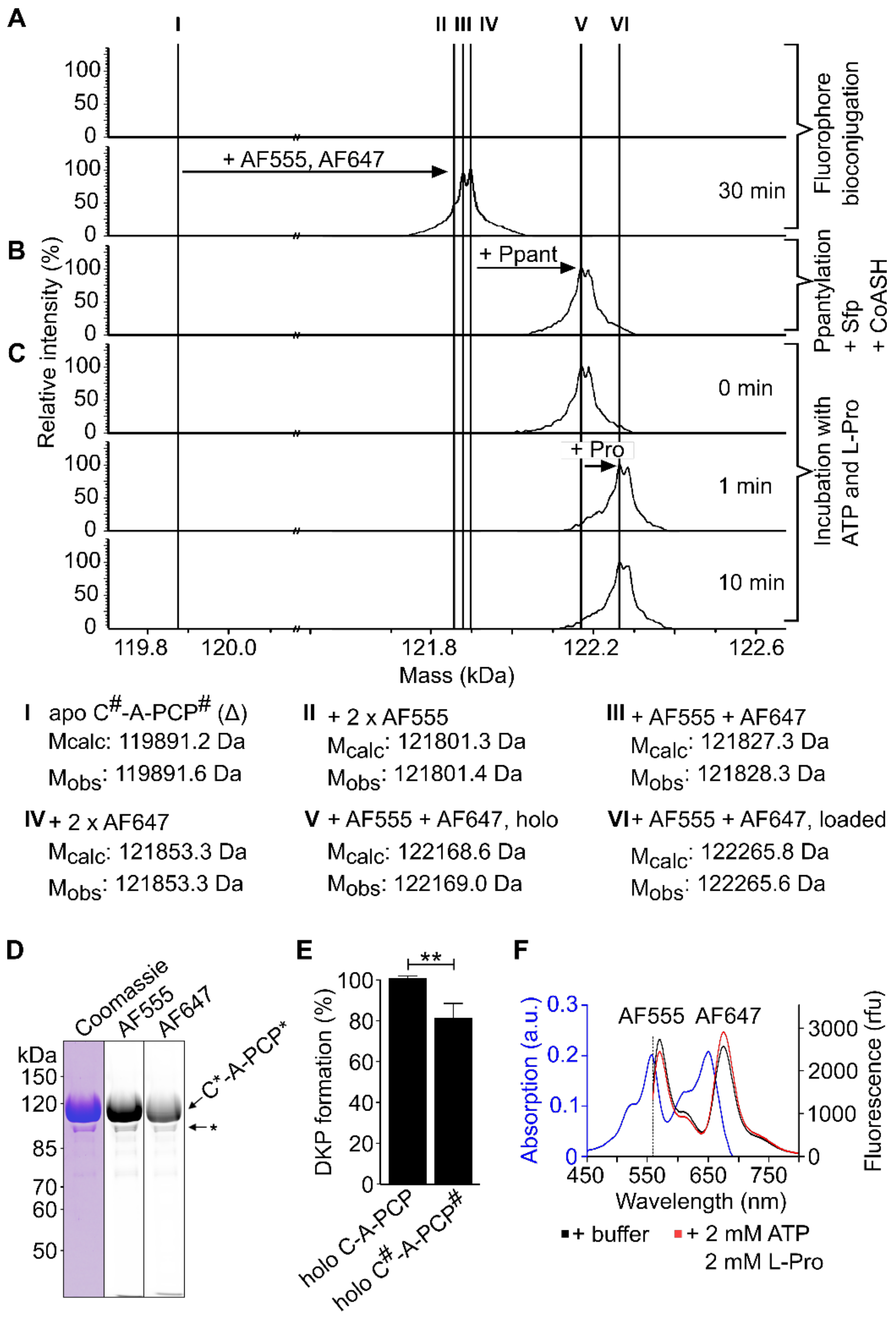

Figure S4. Development of the TycB1 C*-A-PCP* FRET sensor. (A, B, C) ESI-MS analysis of fluorophore bioconjugation, Ppantylation and aminoacylation reaction. (D) SDS-PAGE analysis of AF555&AF647-conjugated C*-A-PCP* by Coomassie-staining and the respective fluorescent channels. The asterisk denotes a minor protein contamination. (E) D-Phe-L-Pro-DKP formation with GrsA after 30 min of the wildtype TycB1 module and its cysteine-mutated variant $C^{\#}$-A-$PCP^{\#}$ used for the sensor design. Data represent mean ± SD of 3 independent replicates. Shown are the mean ± SD of three independent replications. ** indicates $p \leq 0.01$, which was determined by Student's *t*-test. (F) Absorption and fluorescence spectra of labeled C*-A-PCP* sensor. Labeling and loading reactions as well as the DKP formation assay were performed as described in the experimental section. The associated concentration gel and HPLC traces are shown in Figure S5.

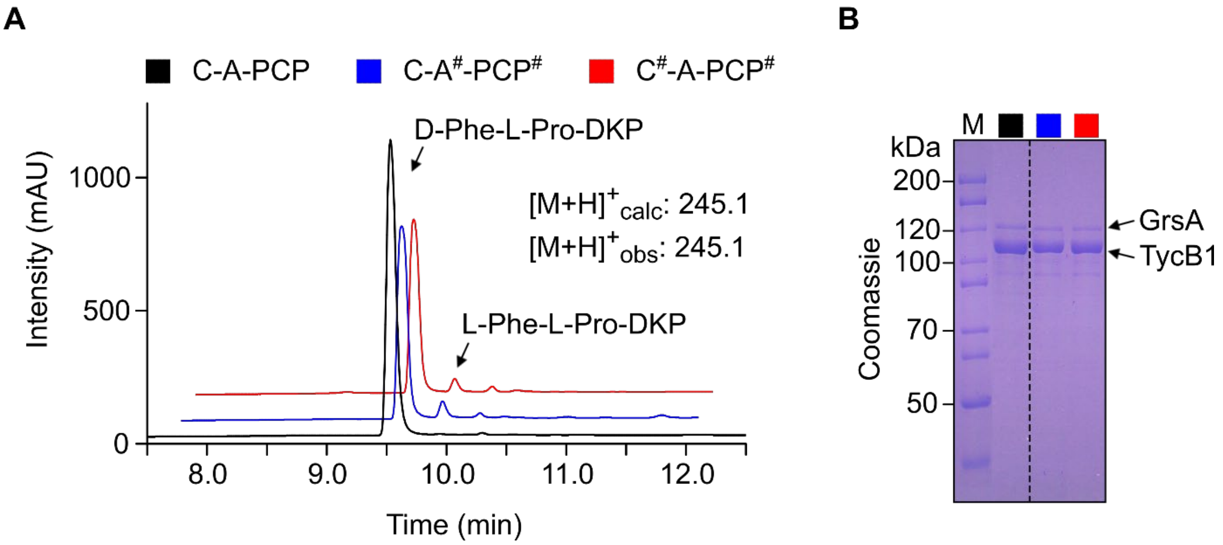

Figure S5. Original data of the D-Phe-L-Pro-DKP formation assay. The dipeptide formation assays using GrsA and one of the shown TycB1 modules were performed as described in the Methods section. (A) HPLC analysis of the cyclic product D-Phe-L-Pro-DKP and L-Phe-L-Pro-DKP. Observed mass corresponds to calculated mass of DKP. (B) Coomassie-stained SDS-PAGE gel of the three TycB1 modules with GrsA investigated. The gel served as a loading control to verify equal concentrations. Shown are representative HPLC traces and SDS-PAGE gels. The assays were performed in triplicate to calculate error bars from standard deviations.

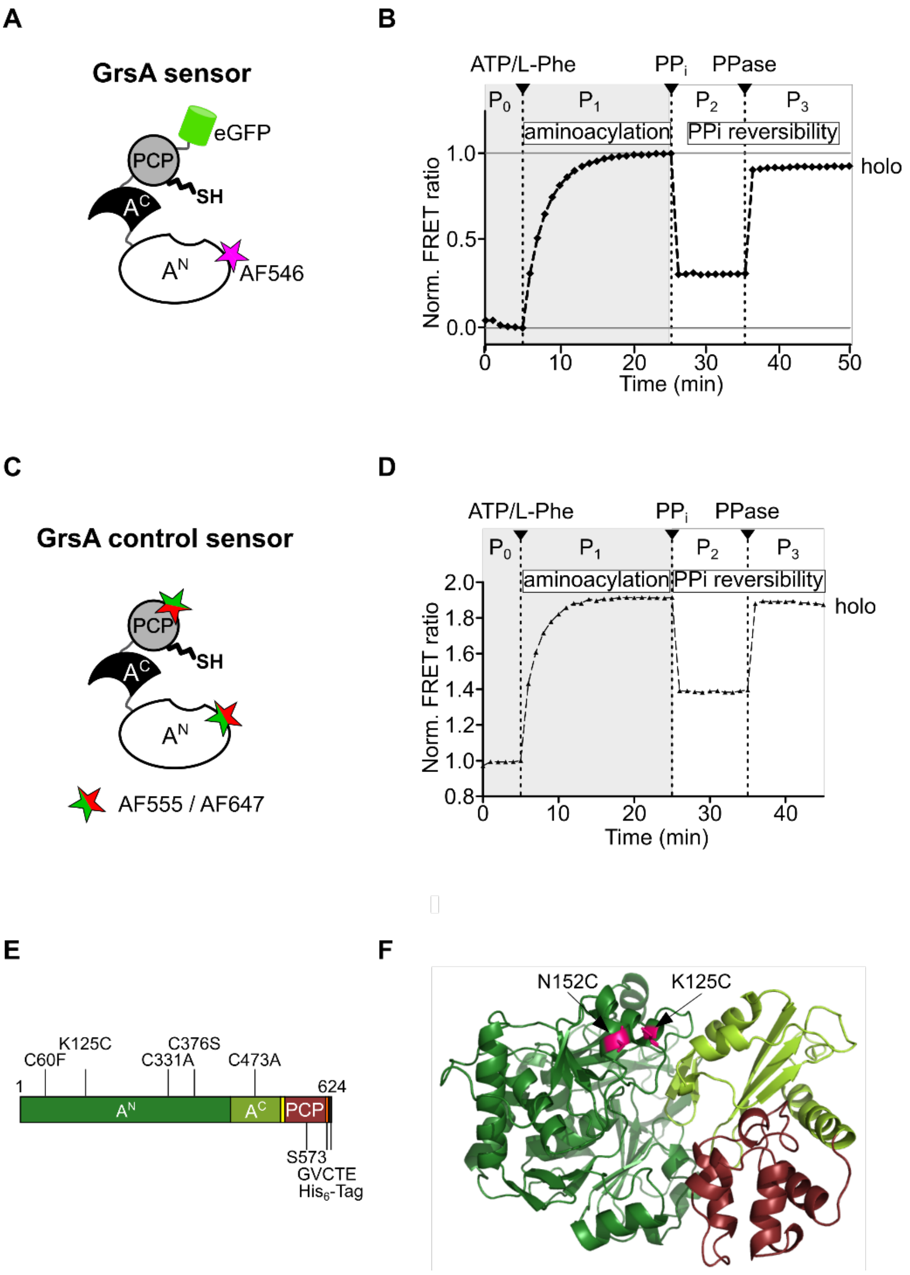

Figure S6. Monitoring conformational changes of GrsA A-PCP FRET sensors in correlation to ligand binding and catalysis. (A, C) Schematic representation of the respective FRET sensor construct. Compared are the previously reported GrsA A-PCP FRET sensor[4] (A) and our new control GrsA A-PCP FRET sensor (C). Each sensor was investigated in holo-form. (B, D) Time-dependent FRET ratio determined after addition at the indicated time points of ATP and L-Phe (each 2 mM), PP_i (2mM (B) and 10 mM (D)) and PPase (2 U). Data shown are from at least 3 biological repeats. Data shown in (B) were taken from previous work.[4] (C, F) Design of the new GrsA A-PCP control sensor shown in (C, D). Since the new TycB1 sensor constructs reported herein utilized a different fluorophore pair and slightly different fluorophore positions compared to the previous GrsA A-PCP sensors,[4, 9] we constructed a new GrsA A-PCP control sensor. This new GrsA A-PCP sensor served as a direct comparison to our new TycB1 C-A*-PCP* and A*-PCP* sensors. To rule out or minimize possible effects of the nature and localization of the donor and acceptor fluorophores on the FRET output, the new GrsA A-PCP sensor was designed in the same way as the respective TycB1 sensors. Instead of a synthetic fluorophore conjugated to N152C and a C-terminally fused eGFP in our previous GrsA A-PCP

sensor designs,[4, 9] we here used stochastic bioconjugation of two cysteines, K125C and a C-terminally appended cysteine in the GVCTE sequence, to attach AF555 and AF647 via maleimide chemistry. K125 was determined as the similarly positioned residue compared to A553 in TycB1 based on a sequence alignment of the GrsA and TycB1 sequences. Likewise, the GVCTE sequence was appended to the respective PCPs in both cases at the same position based on the sequence alignment. (C) and (E) Scheme of the new GrsA A-PCP sensor. (D) Time-dependent change of the FRET ratio as determined for previous GrsA A-PCP sensor designs.[4, 9] (F) Positions of N152C and K125C mapped on the structure of GrsA A-PCP.

Together, these controls suggest that the new GrsA A-PCP FRET sensor design gives a qualitatively very similar FRET read-out as the previous sensor designs. Furthermore, they suggest that differences in the FRET read-out observed for the A-PCP didomain units of the GrsA and TycB1 sensors are not due to the FRET sensor design in terms of the nature of the fluorophores and the attachment positions of the fluorophores. Rather, differences in FRET read-out are likely to stem from structural differences between GrsA and TycB1 A-PCP unit, e.g. in the positioning and orientation of the PCP relative to the A^N subdomain within a certain conformation like the transfer conformation, as well as from possible differences in relative population of individual conformations in the overall conformational equilibrium. Slight differences in positioning and orientation of the PCP relative to the A^N subdomain could for example result in a high-FRET out-put for the Ppant-threading conformation, which is induced by PP_i at excess concentrations in the P2 levels, whereas this conformation does not result in a high-FRET constellation in the GrsA A-PCP sensors.[4]

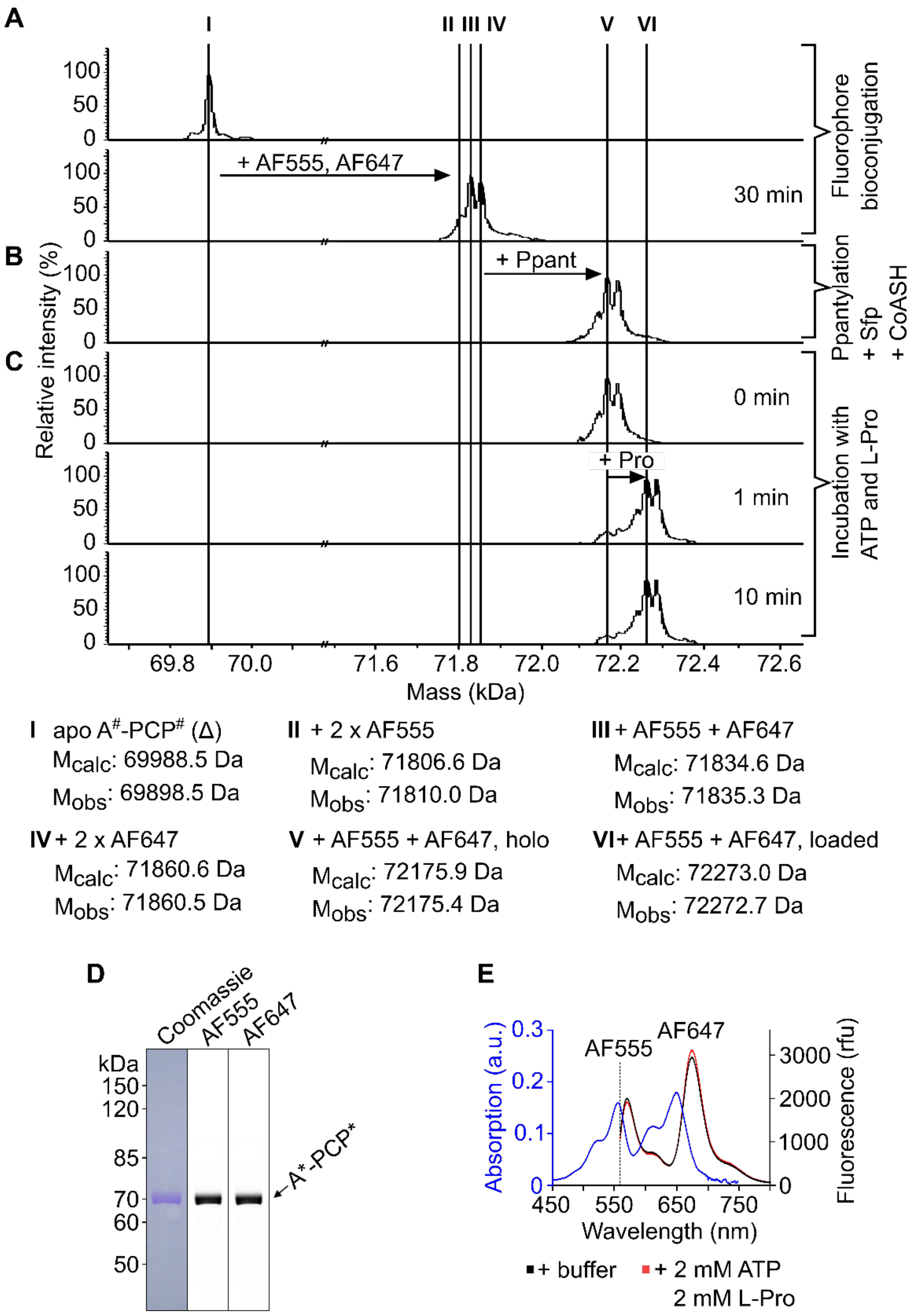

Figure S7. Establishment of the TycB1 A*-PCP* FRET sensor. (A, B, C) ESI-MS analysis of fluorophore bioconjugation, Ppantylation and aminoacylation reaction. (D) SDS-PAGE analysis of AF555&AF647-conjugated C*-A-PCP* by Coomassie-staining and the respective fluorescent channels. The asterisk denotes a minor protein contamination. (E) Absorption and fluorescence spectra of labeled A*-PCP* sensor.

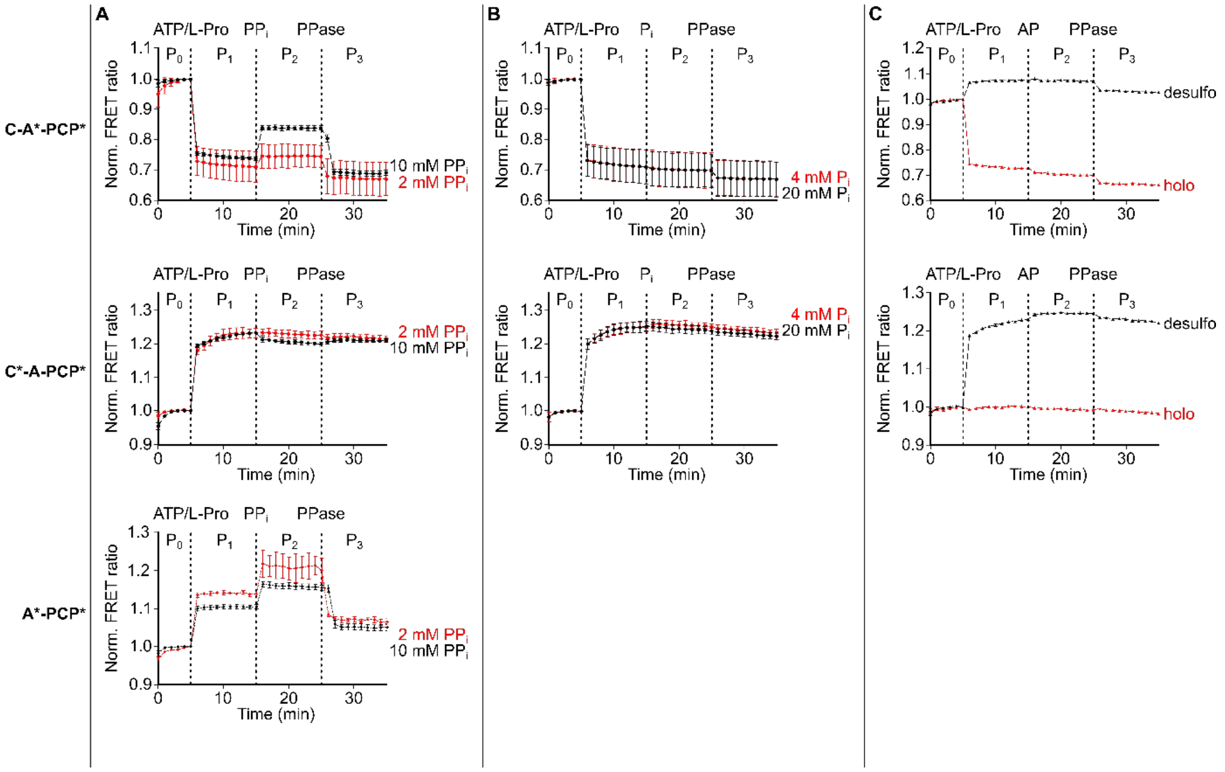

Figure S8. Control experiments to the addition of PP$_i$, P$_i$ and PPase. Shown are normalized data of time-resolved FRET measurements using the TycB1 sensors holo-C-A*-PCP* (top row), holo-C*-A-PCP* (middle row) and the truncated holo-A*-PCP* sensor (bottom row). At the indicated time points, ligands or substrates were added at the following concentrations: ATP and L-Pro (2 mM each), PP$_i$ (2 mM or 10 mM), P$_i$ (4 mM or 20 mM) or PPase (2 U). AP = assay buffer. (A) Control experiments to evaluate the influence of PP$_i$ concentration. (B) Control experiments to evaluate the impact of P$_i$ concentration (which is released from PPase mediated hydrolysis of PP$_i$). (C) Control experiments to evaluate the effect of PPase addition independent of its PP$_i$ hydrolysis activity. Data shown are from 2 biological repeats, each with 3 independent measurements.

These experiments were performed to address differences observed in the interplay of the A-PCP didomain units of the new TycB1 sensors compared to our previously reported GrsA A-PCP sensors. To address the qualitatively opposite response to PP$_i$ in the P2 plateaus, we performed the assays with 2 mM PP$_i$ as an alternative concentration as shown in (A). Both 2 and 10 mM PP$_i$ showed a qualitatively similar effect. To verify that the high concentrations of P$_i$ generated in the PPase reaction had no decisive impact on our observations, the control experiments in (B) were performed. Again, no significant changes were observed, ruling out the possibility that generated P$_i$ could have significantly influenced the read-out of the FRET sensors. Similar findings were made for the control experiments in (C). These experiments show that PPase itself, without any high concentrations of added PP$_i$ that would be converted to P$_i$, does only have a negligible effect on the FRET ratio changes. However, the PPase addition obviously accounted for the differences observed between the P1 and P3 levels. This finding explains why no full reversibility back to the FRET ratio of plateau P1 is observed after PPase addition.

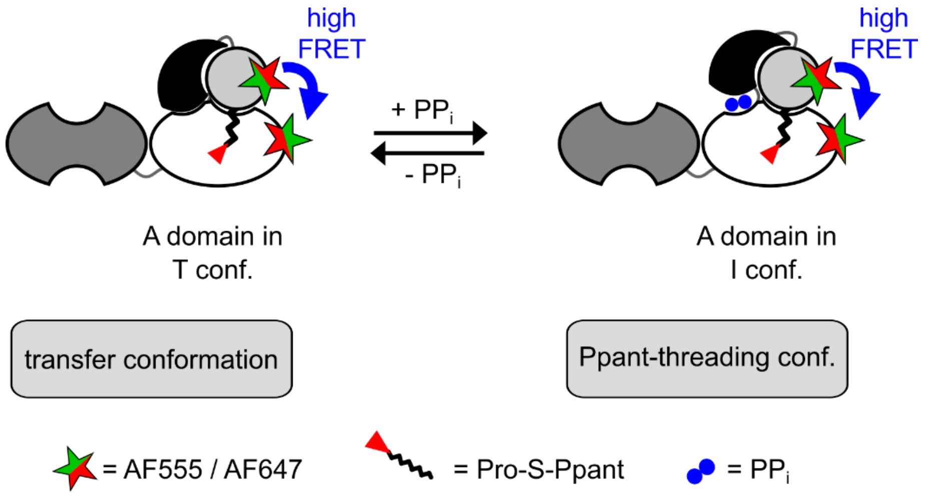

Figure S9. PP$_i$-induced conformational change to the Ppant-threading conformation. Previous work has suggested the existence of at least one intermediary conformation (I conformation) in the domain alternation mechanism between A and T conformations of the A domain.[4] The Ppant-threading conformation was proposed to be an intermediate for the prosthetic group of the PCP to enter and leave the A domain's active site.[4] The biochemical and structural requirements for the Ppant-threading conformation are well represented in an NRPS crystal structure showing the A^N and A^C subdomains of the A domain in a half-open conformation with the acylated Ppant-PCP bound to the active site.[10] In this structure (pdb code 5u89),[10] a binding site for PPi is plausible between the A^N and A^C subdomains.[4] By addition of PP$_i$ at high concentrations (e.g., 2 to 10 mM) this site is likely occupied, resulting in a conformational change from the transfer conformation to the Ppant-threading conformation,[4] as depicted in the Figure. Similar to the transfer conformation of the A-PCP didomain ensemble, the aminoacylated PCP remains bound to the A^N subdomain, consistent with a high FRET ratio for the C-A*-PCP* and A*-PCP* sensors described in Figure 3. The Ppant-threading conformation can thus explain the high FRET ratio of plateau P2 for these sensors in their aminoacylated holo-forms.

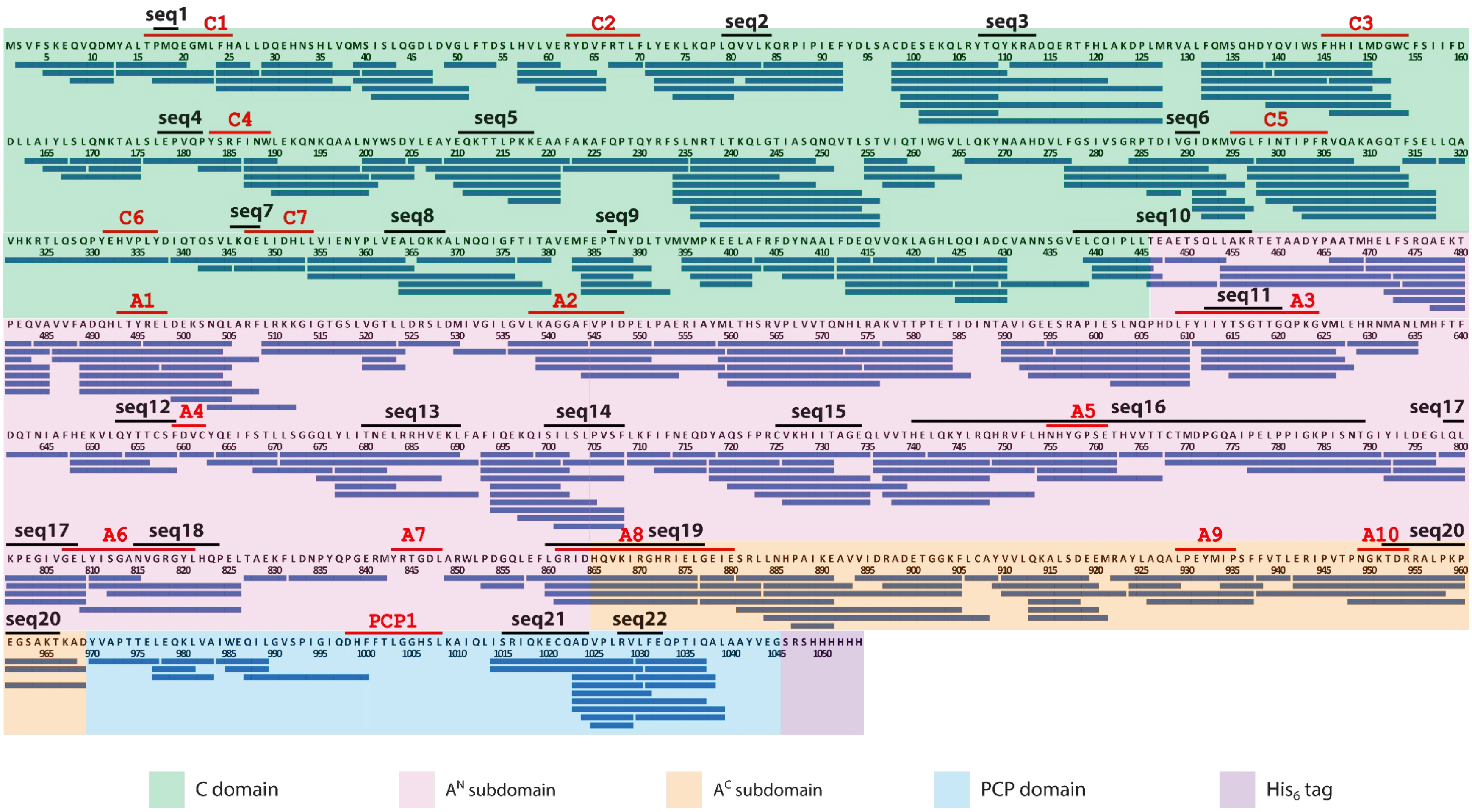

Figure S10. Peptide coverage of full-length holo TycB1 in HDX-MS experiments displayed on the amino acid sequence of the protein. The numbering is according to Uniprot-ID: O30408. Blue bars represent peptides that were identified and analyzed for their HD exchange (HDX). The locations of 22 sequence stretches (seq1 to seq 22, see Supplemental Dataset) that showed altered HDX under the different experimental settings i and ii are shown as black bars. Setting i represents protein in buffer condition and setting ii is protein incubation with ATP and L-Pro. The core motifs C1-C7 in the C domain, A1-A10 in the A domain and PCP1 in the PCP domain according to Marahiel et al.[11] are highlighted in red. The color code of the background refers to the different domains and tag of the protein.

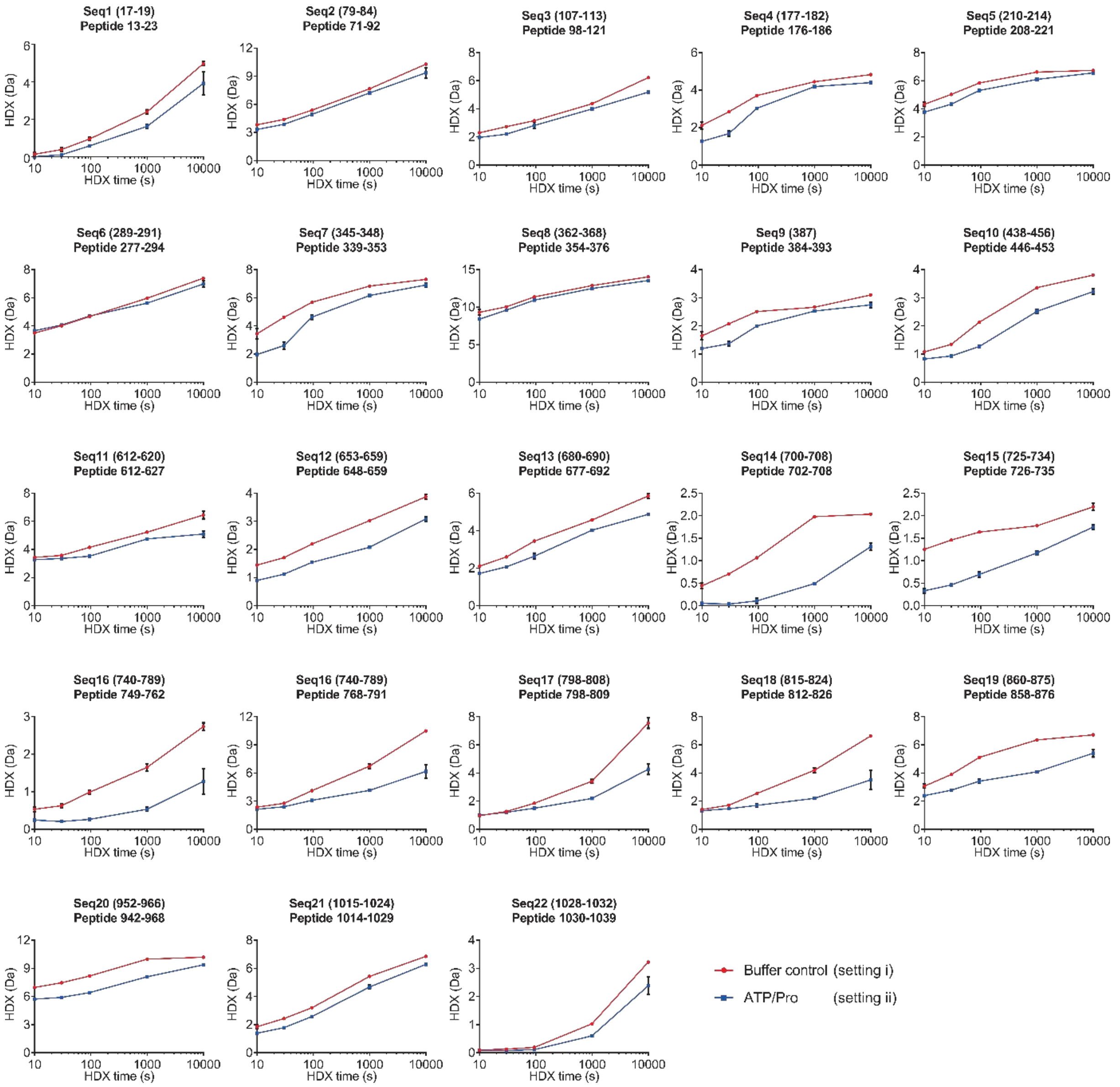

Figure S11. HDX of representative peptides located in the sequence stretches 1-22 (seq1 – seq22), exemplifying the difference in HDX of full-length holo TycB1 between the buffer control (setting i, red trace) and upon incubation with with ATP and L-Pro (setting ii, blue trace). The TycB1 residue range covered by the sequences stretches (in brackets) was determined from overlapping peptides as described in the methods section. One representative peptide for each sequence stretch and its residue range is given except for very long sequence stretch 16, for which two representative peptides are depicted. Data represent the mean ± SD (n=3).

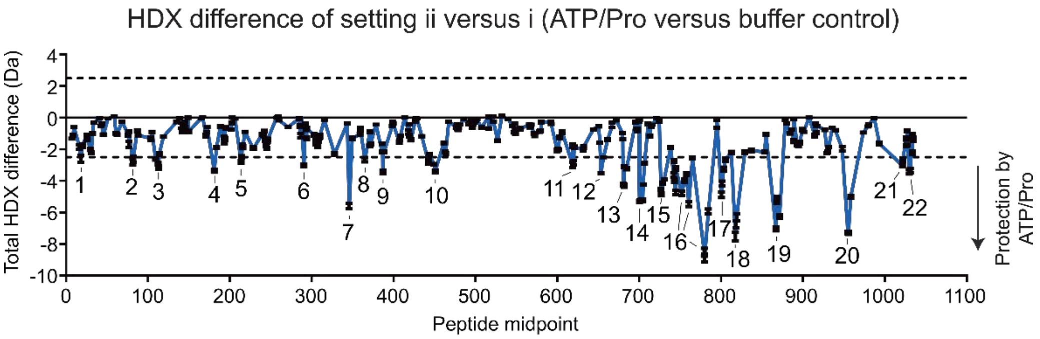

Figure S12. Differences in HDX of holo TycB1 between the settings: buffer control (i) and ATP + L-Pro (ii). Graph depicts the total difference in HDX of setting ii minus setting i. To calculate the total HDX difference, the differences in HDX between settings ii and i were calculated for each time point of incubation in deuterated solvent (i.e. 10, 30, 95, 1000, 10000 s) and then summed up. The graphs depict the mean ± SD (n=3) of the total HDX difference according to the midpoint of the peptides (calculated as sum of the number of the first and last amino acid of the peptide divided by two). The dashed lines indicate the limits of the 95% confidence interval. Numbers denote the sequence stretches (seq) also shown in Figure S10 and Figure S11, which exhibit alterations in HDX above the confidence interval threshold.

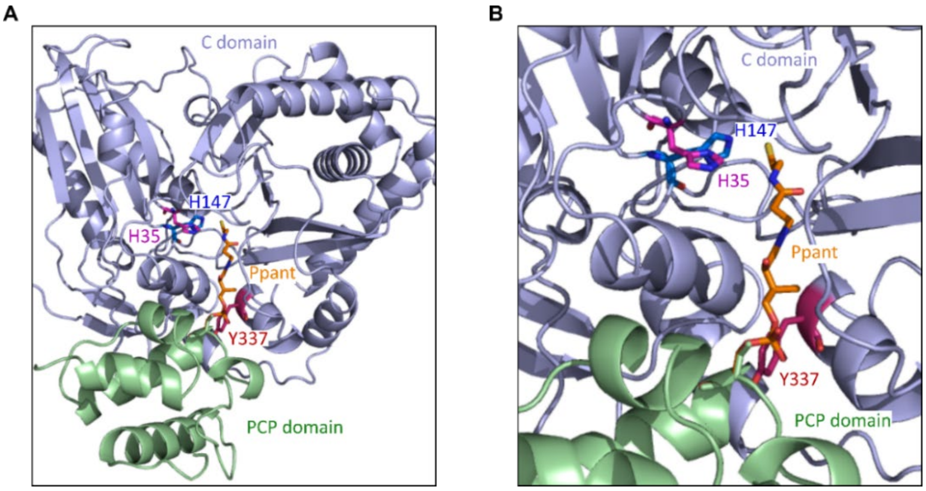

Figure S13. Structural considerations for positioning a photocage group at the acceptor position of the C domain. Depicted is a crystal structure of the C domain catalytic center to visualize the selected ONBY positions. (A) Sequence of TycB1(wt) modeled into the PDB structure 4ZXI,[12] in which the PCP domain is binding the C domain, using Phyre2.[13] Shown is the V-shaped structure of the C domain (grey) with interactive PCP domain (green). ONBY positions H35 (magenta) and Y337 (red) as well as the catalytically relevant H147 (blue) of the HHxxxDG motif are highlighted. S1007 with the Ppant moiety (orange) is also in sticks representation. The A domain is not shown for reasons of clarity. The H35 side chain points to the Ppant moiety, while the Y337 side chain points towards the body of the PCP domain. The photo-labile protecting group of ONBY is expected to result in steric clashes in both cases when the PCP were to productively bind the C domain. As the H35 position is situated closer to the active site than the Y337 position, the respective ONBY mutation might have a more pronounced effect on catalysis once the enzyme is in the C conformation, either by better sterically blocking the Pro-S-Ppant from the active site or by structurally perturbing the active site residues. (B) Close-up view.

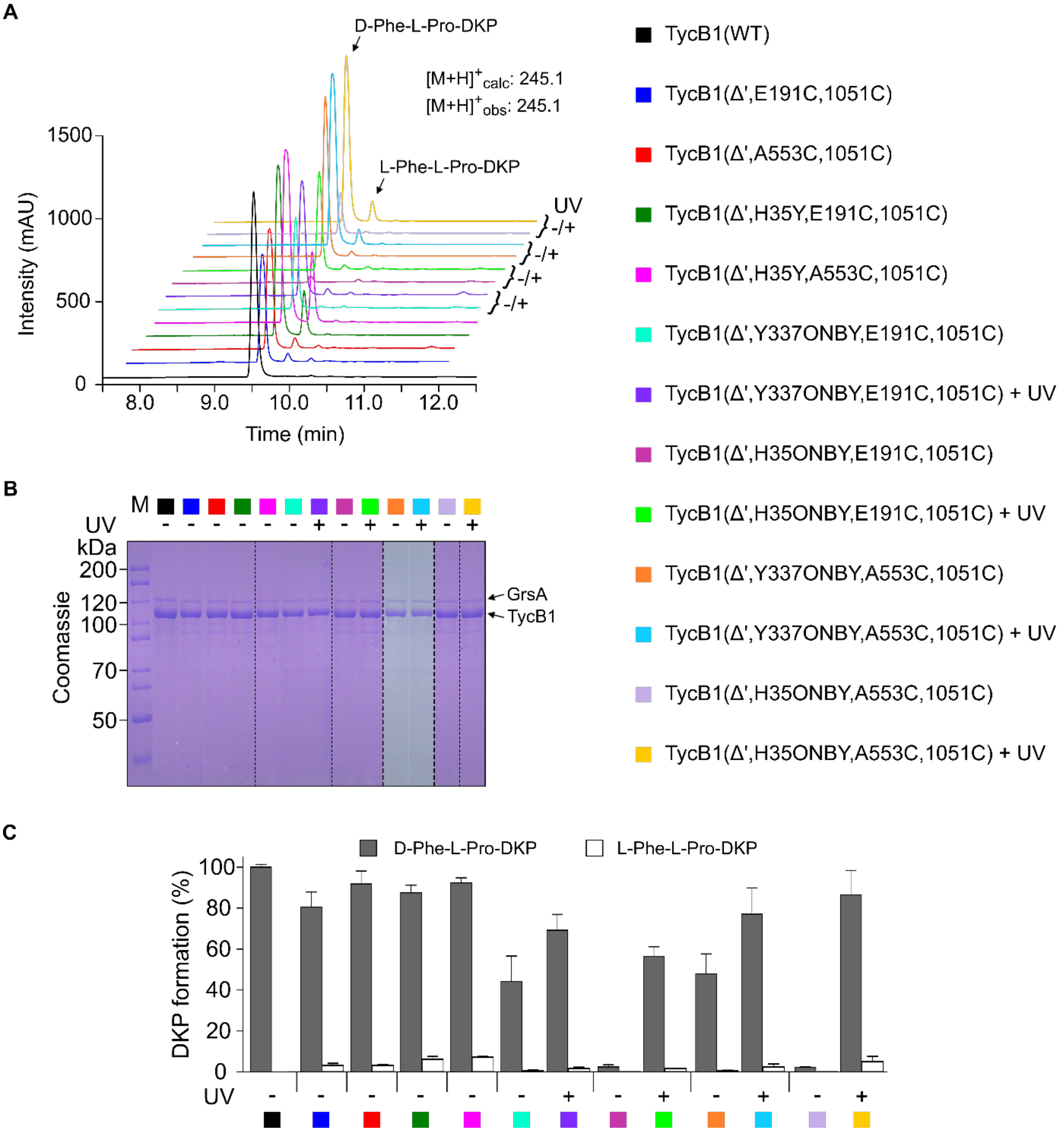

Figure S14. Original data of the D-Phe-L-Pro-DKP formation assay. The dipeptide formation assays using GrsA and one of the shown TycB1 mutants were performed as described in the Methods section. (A) HPLC analysis of the cyclic product D-Phe-L-Pro-DKP and L-Phe-L-Pro-DKP. Observed masses correspond to calculated mass of DKP. (B) Coomassie-stained SDS-PAGE gel of the investigated TycB1 mutants in mixture with GrsA. The gel served as a loading control to verify equal concentrations. Shown are representative HPLC traces and SDS-PAGE gels. The assays were performed in triplicate to calculate error bars from standard deviations. (C) Quantitative analysis of the D-Phe-L-Pro-DKP and L-Phe-L-Pro-DKP products from the HPLC data shown in (A). All product yields were normalized to the 100% of D-Phe-L-Pro-DKP formed by the wild-type TycB1. The TycB1 mutants formed detectable amounts of L-Phe-L-Pro-DKP, with the highest yield of 7.2% for the H35Y mutations. Please note that the levels of L-Phe-L-Pro-DKP were not accounted for in Figure 5 of the main text. The differences of the total DKP amounts to the D-Phe-L-Pro-DKP yields would only marginally affect the activity changes induced by the photo-activation.

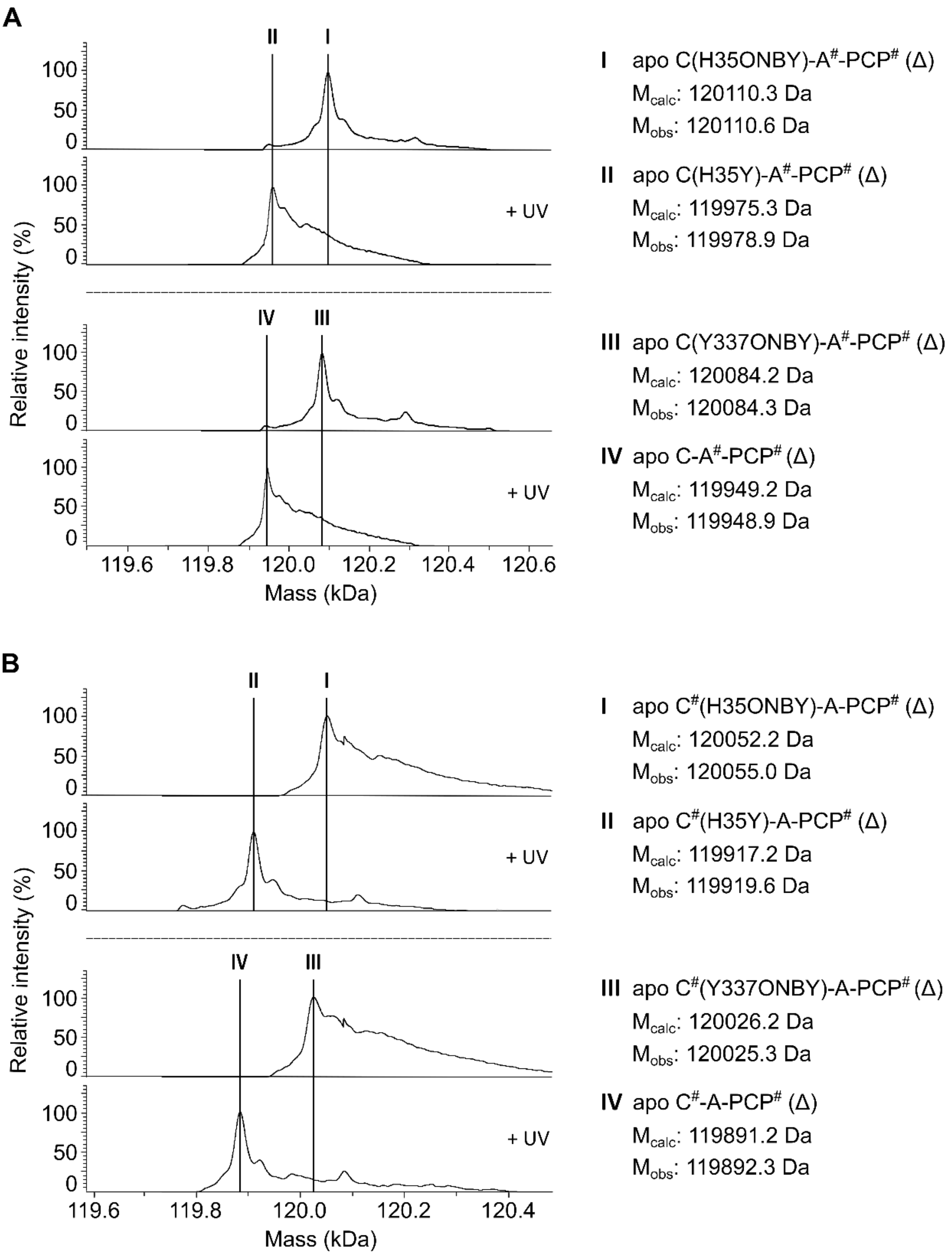

Figure S15. Photo-decaging of TycB1 proteins with ortho-nitrobenzyl-tyrosine (ONBY) examined by ESI-MS. The indicated H35ONBY and Y337ONBY mutants of the purified proteins were irradiated with UV light at 365 nm for 10 sec. ESI-MS spectra were recorded before and after the treatment with light. (A) ESI-MS spectra of the indicated C-A#-PCP#(ONBY) proteins. (B) ESI-MS spectra of the indicated C#-A-PCP#(ONBY) proteins. Calculated and observed masses are listed in the figure.

Supporting References

[1] J. Zettler, H. D. Mootz, *Febs J* **2010**, *277*, 1159-1171.

[2] E. Gasteiger, C. Hoogland, A. Gattiker, S. Duvaud, M. R. Wilkins, R. D. Appel, A. Bairoch, in *The Proteomics Protocols Handbook* (Ed.: J. M. Walker), Humana Press, **2005**.

[3] A. Deiters, D. Groff, Y. Ryu, J. Xie, P. G. Schultz, *Angew Chem Int Ed Engl* **2006**, *45*, 2728-2731.

[4] F. Mayerthaler, A. L. Feldberg, J. Alfermann, X. Sun, W. Steinchen, H. Yang, H. D. Mootz, *RSC Chem Biol* **2021**, *2*, 843-854.

[5] a) R. M. Clegg, *Methods Enzymol* **1992**, *211*, 353-388; b) V. S. Kraynov, C. Chamberlain, G. M. Bokoch, M. A. Schwartz, S. Slabaugh, K. M. Hahn, *Science* **2000**, *290*, 333-337.

[6] a) T. Stachelhaus, H. D. Mootz, V. Bergendahl, M. A. Marahiel, *J Biol Chem* **1998**, *273*, 22773-22781; b) E. Dehling, G. Volkmann, J. C. Matern, W. Dorner, J. Alfermann, J. Diecker, H. D. Mootz, *J Mol Biol* **2016**, *428*, 4345-4360.

[7] M. Osorio-Valeriano, F. Altegoer, W. Steinchen, S. Urban, Y. Liu, G. Bange, M. Thanbichler, *Cell* **2019**, *179*, 1512-1524 e1515.

[8] A. J. Inglis, G. R. Masson, S. Shao, O. Perisic, S. H. McLaughlin, R. S. Hegde, R. L. Williams, *Proc Natl Acad Sci U S A* **2019**, *116*, 4946-4954.

[9] J. Alfermann, X. Sun, F. Mayerthaler, T. E. Morrell, E. Dehling, G. Volkmann, T. Komatsuzaki, H. Yang, H. D. Mootz, *Nat Chem Biol* **2017**, *13*, 1009-1015.

[10] M. J. Tarry, A. S. Haque, K. H. Bui, T. M. Schmeing, *Structure* **2017**, *25*, 783-793 e784.

[11] M. A. Marahiel, T. Stachelhaus, H. D. Mootz, *Chem Rev* **1997**, *97*, 2651-2674.

[12] E. J. Drake, B. R. Miller, C. Shi, J. T. Tarrasch, J. A. Sundlov, C. L. Allen, G. Skiniotis, C. C. Aldrich, A. M. Gulick, *Nature* **2016**, *529*, 235-238.

[13] L. A. Kelley, S. Mezulis, C. M. Yates, M. N. Wass, M. J. Sternberg, *Nat Protoc* **2015**, *10*, 845-858.
